# An interpretable deep learning framework uncovers features governing CRISPR-Cas9 genome-editing efficiency

**DOI:** 10.1093/bioinformatics/btag483

**Published:** 2026-07-02

**Authors:** Nasim Bakhtiyari, Yosef Masoudi-Sobhanzadeh, Safar Farajnia, Sushant Kumar

**Affiliations:** Drug Applied Research Center, Tabriz University of Medical Sciences, Tabriz, Iran; Department of Computer Engineering, Istanbul Rumeli University, Istanbul, Turkey; Department of Molecular Medicine, Faculty of Advanced Medical Sciences, Tabriz University of Medical Sciences, Tabriz, Iran; Drug Applied Research Center, Tabriz University of Medical Sciences, Tabriz, Iran; Biotechnology Research Center, Tabriz University of Medical Sciences, Tabriz, Iran; Princess Margaret Cancer Centre, University Health Network, Toronto, Ontario, Canada; Department of Medical Biophysics, University of Toronto, Toronto, Ontario, Canada

## Abstract

**Motivation:**

CRISPR-Cas9 genome-editing efficiency is strongly influenced by the sequence composition and positional context of single-guide RNAs (sgRNAs). Although numerous deep learning–based models have been developed to predict Cas9 efficiency from sgRNA sequences, most operate as black boxes, offering limited insight into the sequence determinants underlying Cas9 activity. In addition, previous studies often overlook how the positional context of sequence motifs within sgRNAs influences their effects on Cas9 binding or cleavage.

**Results:**

We introduce *DeepCC9*, an interpretable machine learning framework that combines explicit sequence feature extraction with a residual block–based deep architecture to improve interpretability and identify composition- and position-based motifs governing Cas9 genome-editing efficiency. We applied this method to multiple Cas9 variant datasets, achieving superior predictive performance compared with existing methods while enabling direct interpretation of sequence motifs and their positional effects. Our analysis uncovered 74 sequence motifs enriched or depleted at specific positions within sgRNAs and strongly associated with Cas9 efficiency, providing mechanistic insight into sequence features that influence guide performance. Together, these results establish *DeepCC9* as a generalizable and interpretable framework for modeling sequence–function relationships and advancing the understanding of the sequence determinants underlying CRISPR-Cas9 genome editing.

**Availability and implementation:**

The authors have implemented their algorithm in the Python programming language (version 3.X), which is accessible using (https://zenodo.org/records/20073890).

## 1 Introduction

CRISPR-Cas9 is a widely employed genome-editing technology for modifying DNA sequences in living cells (Alsaiari *et al.* 2024). It has a wide range of applications, including correcting disease-causing mutations, inducing mutations to model diseases, systematically perturbing genes to identify potential drug targets, modifying crop genes to improve their quality, and altering specific genes to study their function (Sánchez-Rivera and Jacks 2015). CRISPR-Cas9 relies on the Cas9 enzyme, an RNA-guided nuclease that can be directed to a specific genomic location. This targeting is achieved by a synthetic single-guide RNA (sgRNA), which contains a 20-nucleotide sequence that guides Cas9 to complementary DNA (Cong *et al.* 2013). Despite its advantages, CRISPR-Cas9 can introduce unintended modifications, known as off-target effects, which limit its precision and broader application (Singh *et al.* 2015). To address this issue, several engineered variants have been developed to improve specificity, including enhanced SpCas9 (eSpCas9(1.1)) and high-fidelity Cas9 (SpCas9-HF1) (Kim *et al.* 2020). Meanwhile, experimental studies have shown that the sgRNA sequence influences the genome-editing efficiency of CRISPR-Cas9 (Wang e*t al.* 2019). The genome-editing efficiency of Cas9 variants is often measured as the fraction of productive edits at the intended target site relative to all observed edits. Therefore, careful design of the sgRNA is crucial for successful genome-editing applications, as its sequence-specific features affect genome-editing performance. Indeed, the performance of Cas9-mediated genome editing depends on several factors associated with the sgRNA sequence, which can be considered criteria for ranking and selecting sgRNAs. First, a GC content of 40%–60% is often preferred, since extremely low GC content may reduce sgRNA–DNA hybrid stability, whereas excessively high GC content may promote stable secondary structure formation that interferes with sgRNA function. Second, some homopolymeric motifs, such as TTTT, may cause premature transcription termination during sgRNA expression and consequently reduce genome editing efficiency. Third, hairpin structures formed within the sgRNA may interfere with target recognition and binding of the Cas9–sgRNA complex to the target genomic region. Fourth, the positions of specific sequence motifs within the sgRNA are important, because nucleotides near the PAM-proximal seed region can substantially affect cleavage and knockout efficiency, in some cases by up to 10-fold (Konstantakos *et al.* 2022).

Given the dependence of CRISPR-Cas9 efficiency on the sgRNA sequence, numerous machine learning methods have been developed to predict this efficiency (also referred to as Cas9 genome-editing efficiency) (Lin and Wong 2018, Schaefer *et al.* 2019, Sun *et al.* 2019, Hiranniramol *et al.* 2020, Charlier *et al.* 2021, He *et al.* 2021, Giner *et al.* 2023, Guan and Jiang 2023, Huang *et al.* 2023, Yang *et al.* 2024, Hou *et al.* 2025). These approaches can be broadly categorized into two groups: (i) feature-engineering-based and (ii) deep learning-based methods (Zhang *et al.* 2022, 2023, Toufikuzzaman *et al.* 2024). Feature-engineering-based machine learning methods utilize pre-determined features and employ conventional algorithms such as gradient-boosted regression trees, support vector regression (SVR), XGBoost, and multilayer perceptron (Wang *et al.* 2019, Sherkatghanad *et al.* 2023). While they offer interpretability at single-nucleotide resolution, these models often struggle with large-scale datasets and exhibit limited predictive performance. To address the performance limitations of conventional algorithms, deep learning techniques such as convolutional neural networks (CNNs) and recurrent neural networks (RNNs) have been developed to model Cas9 genome-editing efficiency (Chuai *et al.* 2018, Kim *et al.* 2018). These methods transform sgRNA sequences into one-hot encoded representations and, in the case of CNN, learn hierarchical sequence features through convolutional layers followed by nonlinear activation and pooling operations. Here, a feature refers to a short DNA sequence (SDS) motif that helps the model predict Cas9 editing efficiency with minimal error. Despite their low predictive error rates, deep learning models designed to forecast Cas9 genome-editing efficiency often operate as black boxes, lacking interpretability.

To address the limited predictive accuracy of classical machine learning methods and the lack of interpretability in earlier deep learning models for predicting Cas9 genome-editing efficiency, we introduce an interpretable framework called *DeepCC9*. This framework extracts over 7000 features and selects approximately 500 as candidate contributors to CRISPR–Cas9 genome-editing efficiency. The candidate features are then used as input to the fully connected layers of a deep residual network for prediction. The final set of informative features is determined by analyzing the weights of the first layer of the deep residual network. Compared to previous methods, which often lacked interpretability and mainly focused on composition-based features, the interpretable framework of *DeepCC9* allowed us to identify position-based sequence motifs that earlier studies largely overlooked. We identified 74 composition- and position-based features (informative features) that influence the genome-editing efficiency of three different Cas9 variants, achieving an average mean squared error (MSE) of 0.009. Additionally, we investigated the relationship between these features and nucleosome occupancy and observed statistically significant associations.

## 2 Methods

### 2.1 A general overview

**Figure 1:**
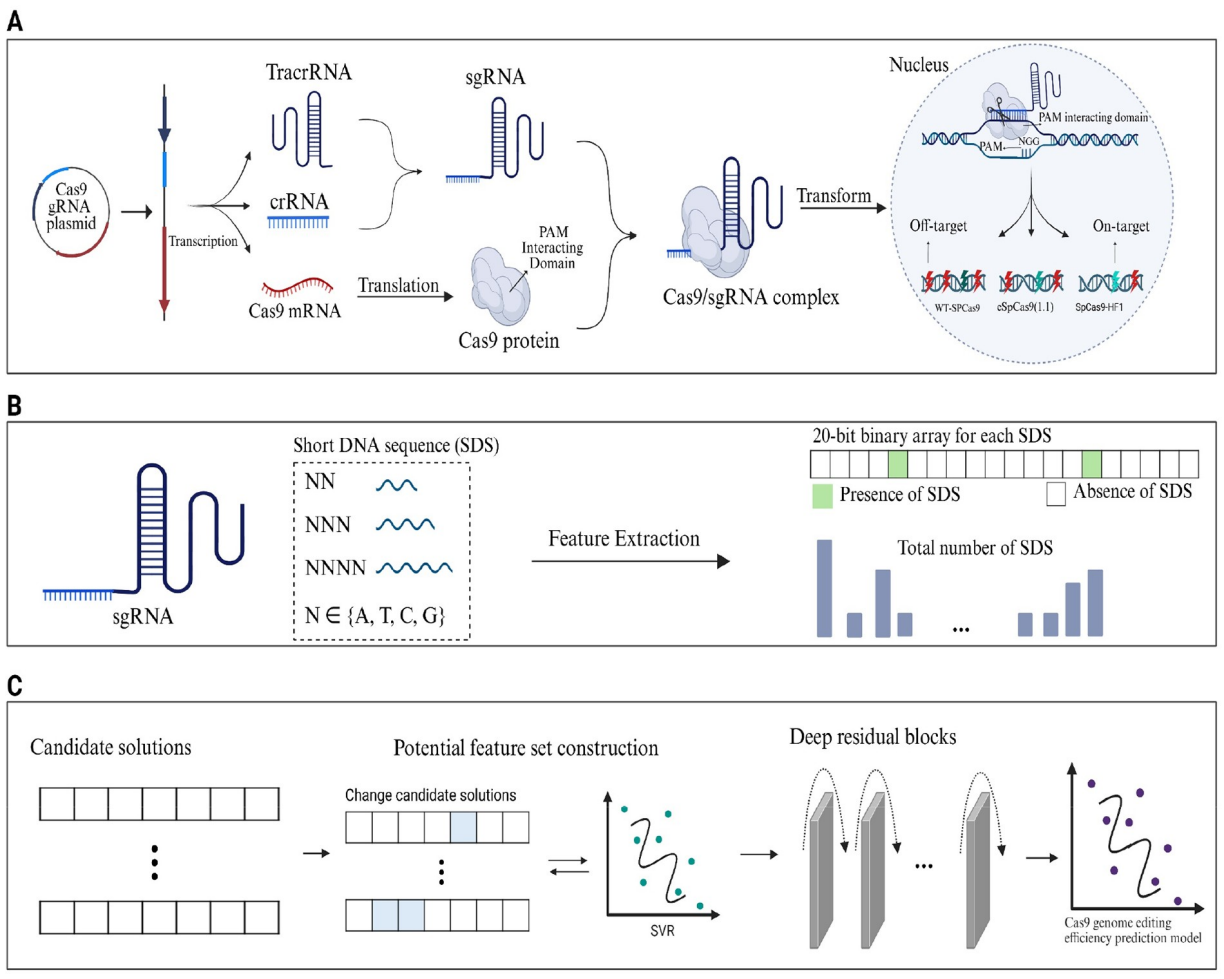
Overview of Cas9-mediated genome-editing and the *DeepCC9* framework. (A) Schematic of Cas9–sgRNA complex formation within the cell, highlighting three Cas9 enzyme variants corresponding to the three datasets used in this study. On-target and off-target genomic regions are indicated in green and red, respectively, with higher green intensity representing increased genome-editing efficiency. (B) Illustration of the feature extraction process. (C) Feature selection procedure and final prediction of genome-editing efficiency by the *DeepCC9* model. PAM: protospacer adjacent motif; SVR: support vector regression.

A typical Cas9-based gene editing method involves a plasmid system engineered to carry both the Cas9 gene and the corresponding sgRNA expression cassettes, which are subsequently delivered into the target cell. Inside the cell, these plasmid components are transcribed into mRNA and then translated into functional Cas9 protein. The crRNA (an 18–20 nucleotide sequence) guides Cas9 to the target DNA via complementary base pairing, while the tracrRNA (a longer RNA with loop structures) serves as a scaffold for Cas9 binding (Jinek *et al.* 2012) (Fig. 1A). For practical gene-editing applications, the crRNA and tracrRNA can be combined into a single guide RNA (sgRNA), enabling precise targeting of nearly any desired genomic sequence. Once the sgRNA binds to Cas9, the resulting Cas9-sgRNA complex translocates into the nucleus, where the sgRNA hybridizes with its complementary target sequence in the genome (Anders *et al.* 2014). In all Cas9 variants, the PAM-interacting domain recognizes the protospacer adjacent motif (PAM) sequence, facilitating guide RNA binding to the target DNA strand.

Similar to our previous work on classifying nucleosome occupancy, our *DeepCC9* method first employs a feature extraction step to obtain features for di-, tri-, and tetra-nucleotide sequences (Masoudi-Sobhanzadeh *et al.* 2024, Masoudi-Sobhanzadeh *et al*. 2025) of sgRNAs for three distinct Cas9 variants (Fig. 1B). Subsequently, *DeepCC9* applies a feature analysis method that integrates the *Trader* optimization algorithm with SVR to identify a set of candidate features that contribute to optimal predictive performance. This candidate feature set is then passed to *DeepCC9’*s deep residual blocks, yielding a genome-editing-efficiency prediction model (Fig. 1C). Finally, the subset of informative features is selected by analyzing the weights associated with the first layer of the trained model.

### 2.2 Architectural differences

Multiple machine learning methods, including those based on deep learning techniques, have been developed to efficiently predict CRISPR-Cas9 sgRNA efficiency and identify sequence features that influence the performance of the CRISPR-Cas9 system (Wang *et al.* 2019, Xiao *et al.* 2021, Zhu *et al.* 2024). However, most of these methods lack interpretability and cannot identify the specific sequence motifs within an sgRNA that drive the models’ predictive performance. For instance, most of these methods can’t ascertain frequencies, and the position of a sequence pattern within the sgRNA affects Cas9 efficiency. Although some methods, such as CapsuleNet, attempt to capture positional information, they are computationally expensive and less practical for large-scale analysis (Sabour *et al.* 2017, Wang *et al.* 2020). To address these limitations, we developed *DeepCC9*, a method inspired by the Deep Residual Networks (ResNet) architecture (He *et al.* 2016) to extract both composition- and position-based features.

**Figure 2:**
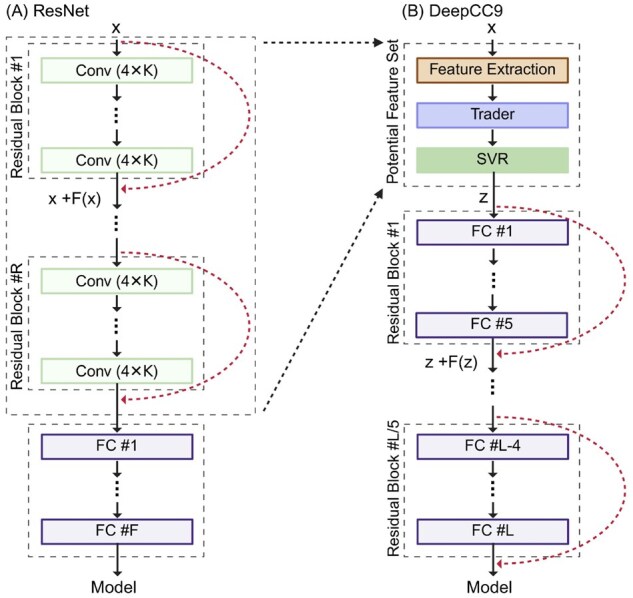
Architectural differences between *DeepCC9* and deep residual networks (ResNet). Red dashed arrows indicate skip connections. (A) Schematic of a standard ResNet architecture, in which skip connections add the input of a residual block to its output within convolutional layers. Here, *K* denotes the kernel length, and the four input channels correspond to the nucleotides A, T, C, and G. (B) Schematic of the *DeepCC9* architecture, where convolutional layers are replaced by explicit feature extraction and feature analysis modules. In this architecture, skip connections are incorporated into the fully connected layers (FC) rather than the convolutional layers.

In particular, the convolutional and pooling layers are replaced with explicit feature-extraction and candidate-feature-selection steps in the *DeepCC9* model architecture (Fig. 2). In addition, skip connections, originally designed to add the input of a residual block to its output, were incorporated into the fully connected layers. This allows *DeepCC9* to combine the predictive power of deep learning with the strong exploration capabilities of metaheuristic algorithms to identify composition- and position-based informative features that influence Cas9 genome-editing efficiency.

#### 2.2.1 Dataset used in this study

We used three datasets from prior studies to train and evaluate our model (Li *et al.* 2023), each corresponding to a different Cas9 variant, as described in Table 1. These datasets were experimentally generated by applying three Cas9 variants and measuring the total number of cases in which each variant successfully edited the target DNA sequence (Wang *et al.* 2019). Consequently, in these datasets, the genome-editing score ranges from 0 to 1, representing the fraction of successful genome-editing events.

**Table 1:** Comparison of three Cas9 variants.

Comparison criterion	WT-spCas9 (WT)	eSCas9(1.1) (ES)	SpCas9-HF1 (SP)
PAM	NGG	NGG	NGG
Off-target editing	High	Reduced	Very low
On-target activity	High	Slightly lower	Variable
Mechanism	Native	Less DNA strand binding	Fewer non-specific contact
Dataset size (#sgRNA)	55 604	56 888	58 617

### 2.3 Feature extraction

We defined a comprehensive set of sequence patterns and extracted features for each pattern. In total, we defined 336 sequence patterns (Equation 1). For each sequence pattern within an sgRNA sequence, three groups of features were extracted (Fig. 1B). Sequence patterns that are significantly enriched or depleted in our analysis are hereafter referred to as *sequence motifs*.


(1) Sequence patterns={N| N∈ {A, C, G, T} and 2≤|N|≤4}

The first group of features quantifies the total number of occurrences of each sequence pattern within an sgRNA (Equation 2), as previous studies have shown that the frequency of specific sequence patterns can influence the Cas9-based genome-editing efficiency (Labun *et al.* 2019).


(2) $$\mathrm{Occurrence}(\mathrm{SeqP},\;\mathrm{sgRNA})=\sum_{i=1}^{L-k+1}\delta (\mathrm{sgRNA}[i:i+k-1],\;\mathrm{SeqP})$$

Where SeqP denotes a sequence pattern, and δ(a,b)=1 if *a* = *b*, and 0 otherwise. The parameter k represents the length of the sequence pattern and can take values of 2, 3, or 4.

The second group of features detects whether each sequence pattern is present or absent at specific positions within an sgRNA. This information is represented as a binary vector of 20, corresponding to the 20 nucleotide positions of the sgRNA. The third group of features comprises four PAM sequences, encoded as a 4-bit binary vector to assess their effects on Cas9 genome-editing efficiency. In total, we extracted 7060 features for each sgRNA.

### 2.4 Selection of candidate features set

Here, candidate feature set construction refers to selecting a large subset of the extracted features that enables our model to predict the genome-editing efficiency of Cas9 with minimal error (Fig. 1C). Since identifying an optimal set of features is computationally expensive and classified as a non-deterministic polynomial problem, a variety of heuristic and metaheuristic methods have been proposed (Mostafa *et al.* 2024). Several studies have shown that combining metaheuristic algorithms with machine learning techniques can lead to the selection of more effective feature subsets, as these methods evaluate features based on the model’s predictive performance (Masoudi-Sobhanzadeh *et al.* 2021, Chaudhuri 2024). In particular, the *Trader* metaheuristic algorithm has been shown to avoid local optima more effectively than other algorithms by incorporating a diverse set of operators (Equations 4–6) (Masoudi-Sobhanzadeh *et al.* 2019). Therefore, we employed a combination of *Trader* and the support vector regression (SVR) to select a subset of effective features for predicting Cas9 genome-editing efficiency. We opted for SVR because it provides a good balance between execution time and prediction error. Overall, the proposed method begins by randomly generating a set of candidate solutions (CS) and distributing them into different groups. For each candidate solution, a prediction model is constructed using SVR, and its fitness is evaluated using MSE (Equation 3). Within a predefined number of iterations, each candidate solution is updated using Equations 4–6, provided that the change reduces the MSE.


(3) $$\mathrm{MSE}=\;\frac{\sum_{i=1}^{n}{\left(Y_{i}-{\hat{y}}_{i}\right)}^{2}}{n}$$

Where *n* is the total number of sgRNAs in a dataset, and $Y_{i}$ and ${\hat{y}}_{i}$ represent the actual and predicted efficiency values of Cas9, respectively.


(4) $$\underset{j\in R^{i}}{⋁}{CS}_{i,G}^{j}=M{CS}_{G}^{j}$$

(5) $$\underset{j\in R^{c}}{⋁}{CS}_{c,G}^{j}=\mathrm{Random}(1,\;F)$$

(6) $$\underset{j\in R^{k}}{⋁}{\mathrm{MCS}}_{k}^{j}=M{CS}_{G}^{j}$$

Here, ${CS}_{i,G}^{j}$ denotes the *j*th variable of the *i*th candidate solution from the *G*th group. *Ri* denotes a set of randomly selected variables from the *i*th candidate solution, and $M{CS}_{G}^{j}$ represents the *j*th variable of the best candidate in the *G*th group. The function *Random* generates a random integer between 1 and the total number of extracted features, denoted by *F*.

Finally, a candidate feature set is constructed by unifying all identified features across all candidate solutions (Equation 7):


(7) $$\mathrm{PotentialFeatureSet}=\overset{n_{CS}}{\underset{i=1}{\cup }}\overset{n_{V}}{\underset{j=1}{\cup }}{CS}_{i}^{j}$$

Where $n_{\mathrm{CS}}$ and $n_{V}$ denote the total number of candidate solutions and the number of variables in each candidate solution, respectively. Here, ${\mathrm{CS}}_{i}^{j}$ denotes the jth variable of the ith candidate solution.

To prevent overfitting and avoid information leakage during candidate feature set selection, all steps of feature extraction and selection using SVR and Trader were performed strictly within each fold of the 5-fold cross-validation. That is, features were selected using only the training data in each fold, and the test data were never used during feature selection or model training. Additionally, the algorithm was stopped before full convergence, and a candidate subset of informative features was selected.

### 2.5 Generation of the prediction model

Although SVR can generate a prediction model for Cas9 genome-editing efficiency, state-of-the-art deep learning methods often achieve better performance. Therefore, we constructed the final prediction model using deep residual blocks, which mitigate the vanishing gradient problem by adding the input of the rth residual block to its output (Equation 8). This process begins after selecting candidate solutions using the method described in the previous section. In our implementation, each residual block consists of five fully connected layers, and the input to the first layer comprises all features included in the candidate feature set defined in Equation 7.


(8) Outji={Inpji−4+δ(∑k=1NOutki−1×Wk,ji−1) if i is divisible by 5δ(∑k=1NOutki−1×Wk,ji−1) Otherwise 

Where ${\mathrm{Inp}}_{j}^{i}$ and ${\mathrm{Out}}_{j}^{i}$ represent the input and output of the *j*th neuron in the *i*th layer, respectively. $W_{k,j}^{i-1}\;$denotes the weight of the edge connecting the *k*th neuron of the (*i-1)*th layer to the *j*th neuron of the *i*th layer. δ represents the sigmoid activation function. The number of epochs and the batch size were set to 1000 and 200, respectively. To prevent overfitting, an early stopping strategy was employed with a patience value of 5. During training, a snapshot of the model was saved at each iteration. After training stopped, the model from the fifth-to-last iteration was retrieved and used as the final CRISPR-Cas9 genome-editing efficiency prediction model.

### 2.6 Selection of the final subset of features

After constructing the Cas9 genome-editing efficiency prediction model using deep residual blocks, the final subset of features was selected by analyzing the weights of the first fully connected layer in the residual blocks, following an approach similar to that described in a previous study (Angermueller *et al.* 2017). To this end, the set of informative features (final subset of features) was defined as:


(9) $$\text{InformativeFeatures}=\{{\mathrm{PF}}^{i}\;\;|\;\;|\sum_{k=1}^{n_{\mathrm{PF}}}\left({\mathrm{Inp}}_{i}^{1}\times W_{i,k}^{1}\right)|>\mu \}$$

Where the threshold μ is defined as:


(10) $$\mu =\;\frac{\sum_{j=1}^{n_{\mathrm{PF}}}\left|\sum_{k=1}^{n_{\mathrm{PF}}}\left({\mathrm{Inp}}_{j}^{1}\times W_{j,k}^{1}\right)\right|}{n_{\mathrm{PF}}}$$

Here, ${\mathrm{PF}}^{i}\;$denotes the ith feature in the candidate feature set, and ${\mathrm{Inp}}_{i}^{1}$ represents its value at the input of the ith neuron in the first fully connected layer. In addition, $n_{\mathrm{PF}}$ denotes the total number of features in the candidate feature set, and $W_{i,k}^{1}$ represents the weight connecting the ith neuron in the first fully connected layer to the kth neuron in the second fully connected layer.

### 2.7 Association between informative features and nucleosomes

Nucleosomes play a crucial role in regulating DNA accessibility and may therefore affect the genome-editing efficiency of Cas9. To assess the relationship between informative features and nucleosome positioning, we performed Fisher’s exact test. Specifically, we investigated the enrichment or depletion of informative features within nucleosomal DNA.

We used MNase-seq fragment data from seven human lymphoblastoid cell lines to identify dyad positions across the human genome, indicating nucleosome positioning. These MNase-seq data represent the highest-resolution dataset available to our knowledge (Gaffney *et al.* 2012). To generate the nucleosome occupancy profile, 147 bp fragments (GSE36979) were mapped to the hg38 reference genome, as this fragment size likely corresponds to nucleosomal DNA, and their midpoint nucleotide may accurately indicate dyad positions (Masoudi-Sobhanzadeh *et al.* 2024). The resulting nucleosome occupancy profile was then smoothed, and the nearest peaks to local smoothed maxima were considered as dyad positions (Peng *et al.* 2024). Since nucleosome positioning varies across cell lines, we filtered dyad positions to retain only well-positioned nucleosomes with a normalized stringency score greater than 0.5, similar to a previous study (Valouev *et al.* 2011). These nucleosomes typically correspond to positions that are consistently occupied at the same genomic location across the cell population, and some of them are considered cell-type independent(Gaffney *et al.* 2012, Buitrago *et al.* 2019). To examine the association between the identified features and nucleosomes (using Fisher’s exact test), the human genome was divided into two categories: (i) nucleosomal regions, and (ii) inter-nucleosomal regions, defined as the DNA segments connecting two nucleosomes that do not overlap with any nucleosomal regions (hereafter referred to as non-nucleosomal DNA). To avoid potential biases, we further filtered the data to include only non-repetitive genomic regions, as indicated by lowercase characters in the genome annotation.

## 3 Results

### 3.1 Comparing *DeepCC9* with previous studies

Since constructing the interpretable feature set is a crucial step in our model development, we conducted a systematic evaluation comparing various optimization algorithms for feature selection and their impact on the predictive performance of our model (*DeepCC9*). Then, we compared the evaluation results of our best-performing *DeepCC9* with those of other methods for predicting Cas9 genome-editing efficiency, using criteria commonly used to assess deep learning models.

**Figure 3:**
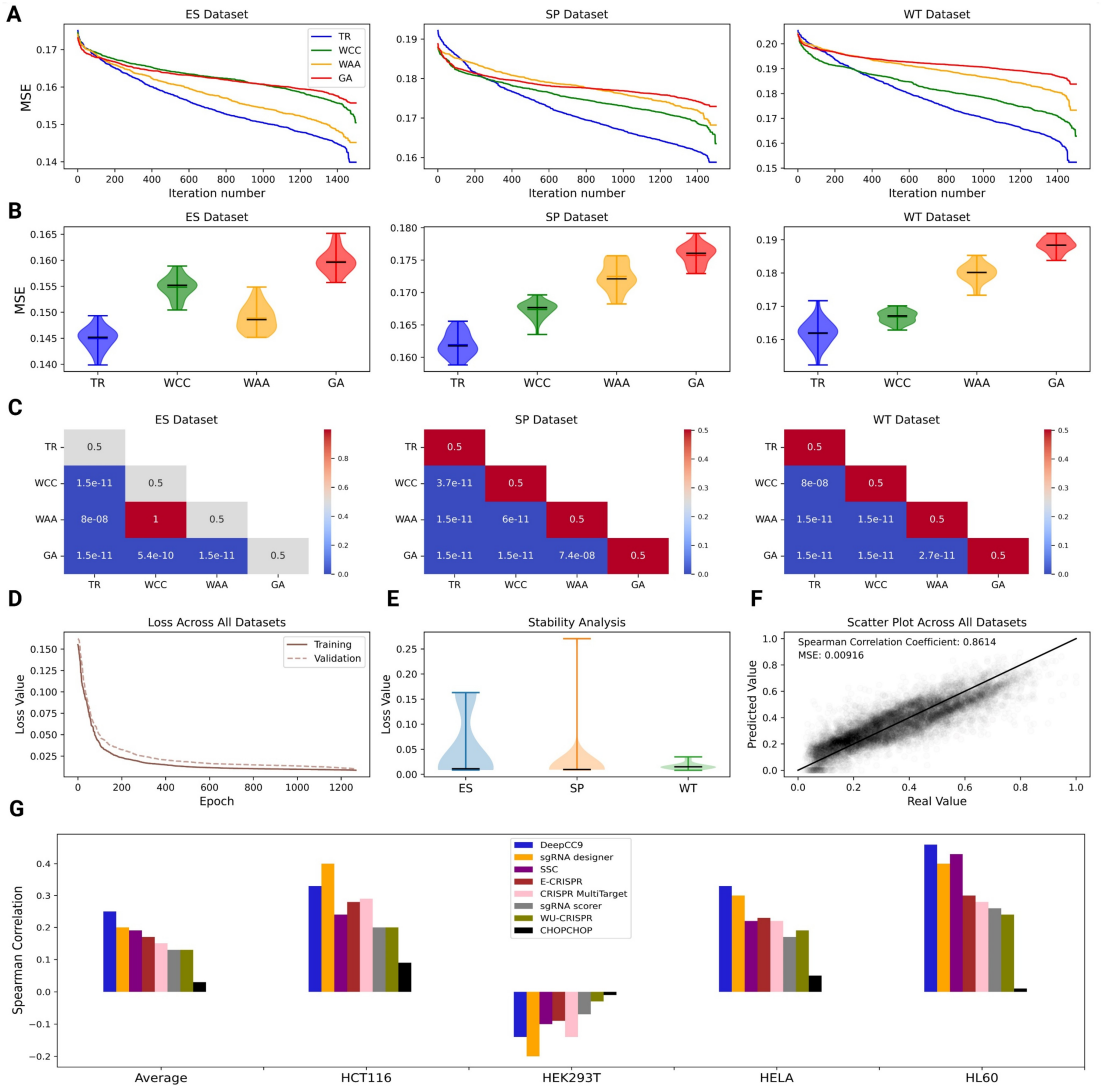
Evaluation of *DeepCC9* from a computational perspective. Part *A* shows the convergence behavior of the optimization algorithms used for candidate feature set construction, and Part *B* shows their stability across 50 independent runs. Part *C* presents the statistically significant differences in the MSE distributions of the algorithms. Parts *D, E, and F* assess the performance of the final Cas9 genome-editing efficiency prediction model based on various deep learning-related criteria. Part *G* represents the Spearman correlation obtained by applying models trained on a specific dataset to independent datasets. ES: eSCas9(1.1) dataset; GA: genetic algorithm; MSE: mean squared error; SP: SpCas9-HF1 dataset; TR: *Trader* algorithm; WAA: weighted average algorithm; WCC: world competitive contests algorithm; WT: WT-spCas9 dataset.

We evaluated the performance of four distinct optimization algorithms for constructing the potential feature set in our *DeepCC9* framework: the *Trader* (TR) algorithm, genetic algorithm (GA), world competitive contests (WCC), and weighted average algorithm (WAA) (Masoudi-Sobhanzadeh and Motieghader 2016, Cheng and De Waele 2024). These distinct optimization strategies were applied to the extracted features, and the fitness of each candidate solution was evaluated using SVR. Our systematic evaluations indicated that the TR-based optimization method yields the lowest MSE value (mean score of ∼0.145) compared to the other three optimization-based methods. Additionally, the TR-based *DeepCC9* model exhibited a better convergence profile than the other three algorithms (Fig. 3A). All algorithms were allowed the same number of fitness function calls (i.e. SVR evaluations) and were terminated once no further reduction in MSE was observed. Moreover, due to the stochastic nature of the algorithms, each was run 50 times to assess stability. The comparison (Fig. 3B) shows that TR is more stable than the others, with an average MSE of approximately 0.16 across all datasets. Additionally, to assess the statistical significance of differences in the MSE distributions across 50 runs, we performed the Mann–Whitney *U* test with alternative='greater’ (Fig. 3C). The results confirmed significant differences between TR and the other algorithms, further supporting the superiority of the TR-based approach. Here, the null hypothesis assumes that the two algorithms perform similarly in selecting Cas9-efficiency-related features. In Fig. 3C, the algorithms on the x- and y-axes are referred to as algorithms A and B, respectively. A *P*value of 1 suggests that the performance of algorithm B is better than that of algorithm A (as observed for WCC and WAA on the ES dataset). A *P*value of 0.5 indicates that the two algorithms perform equivalently. In contrast, a *P*value close to 0 implies that algorithm A consistently and significantly outperforms algorithm B at the 1% significance level. We found that TR, with an average *P*value of approximately 2.23 × 10^−10^, significantly outperformed the other three algorithms in selecting informative features that influence Cas9 performance (Fig. 3B and C).

The extracted data were filtered using the candidate feature set identified by the TR algorithm, and the final Cas9 genome-editing efficiency prediction model was built using *DeepCC9*'s fully connected layers. Figure 3D presents an evaluation of *DeepCC9* across all datasets. A closer inspection of the training and validation loss of our TR-based model indicated no overfitting and improved generalizability for predicting Cas9 genome-editing efficiency (Fig. 3D). Furthermore, we assessed the stability of *DeepCC9* as the parameters associated with our model are randomly initialized (Fig. 3E). To this end, we performed 50 independent runs of *DeepCC9*, resulting in an average MSE of 0.009. Additionally, we correlated experimental Cas9 editing efficiency with predicted efficiency and observed a strong positive correlation (0.86), confirming the model’s ability to accurately predict Cas9 genome-editing efficiency (Fig. 3F).

The generated Cas9 genome-editing efficiency prediction model was compared with prior studies using the Spearman correlation coefficient (SPE) and MSE metrics (Table 2). Since increasing the training set size and reducing the test set size can artificially inflate the algorithm’s performance, we used a 5-fold cross-validation scheme instead of 10-fold cross-validation to provide a more standardized evaluation of our method. The average MSE × 10³ and Spearman correlation coefficient of *DeepCC9* across all datasets were 9.61 and 0.862, respectively. Although *DeepCC9* slightly outperforms other techniques, its superiority over prior studies lies in its ability to identify features that affect the genome-editing efficiency of Cas9. Additionally, *DeepCC9* predicts Cas9 genome-editing efficiency using a minimal set of features, whereas other methods convert sgRNA sequences into one-hot encoded representations and use them directly for prediction, thereby incurring higher computational requirements. The results shown in Table 2 are derived from findings reported in previous studies and from those obtained in the present study.

**Table 2:** Comparison of the proposed method with previous methods.

Method name	Method description	Validation method	Interpretability technique	WT-SpCas9 (WT)		eSpCas9 (1.1) (ES)		SpCas9-HF1 (SP)	
				SPE	MSE × 10^3^	SPE	MSE × 10^3^	SPE	MSE × 10^3^
*DeepCC9*	An interpretable machine learning method based on sequence features and concepts from deep residual networks	5-fold cross-validation	Analysis of the first layer of the deep residual network	0.861	**9.15**	**0.860**	10.9	**0.867**	**9.61**
XGBoost	Feature extraction based on concepts from game theory, followed by model construction using the XGBoost algorithm (Wang *et al.* 2019)	10-fold cross-validation	Not reported	0.845	11.7	0.831	11.5	0.818	13.5
MLP	Transformation of sgRNA sequences into one-hot encoded representations and construction of a prediction model using a multilayer perceptron (Wang *et al.* 2019)	10-fold cross-validation	Not reported	0.842	11.7	0.846	10.5	0.844	11.2
CNN	Transformation of sgRNA sequences into one-hot encoded representations and construction of a prediction model using a convolutional neural network (Wang *et al.* 2019)	10-fold cross-validation	Not reported	0.846	11.3	0.831	11.3	0.834	12.0
RNN	Extraction of biological features based on contribution values and construction of a prediction model using recurrent neural networks (Wang *et al.* 2019)	10-fold cross-validation	Evaluation of handcrafted biological features using SHAP values	0.856	10.4	0.849	10.2	0.851	10.6
TAC	A prediction model based on a temporal attention module capturing the ordering of sequence features (Xiao *et al.* 2021)	Single split	Not reported	0.857	10.3	0.844	10.5	0.851	10.7
SpAC	A prediction model based on a spatial attention module capturing positional information of sequence features (Xiao *et al.* 2021)	Single split	Not reported	0.862	10.1	0.854	9.93	0.857	10.2
EnAC	A prediction model that stacks temporal and spatial attention modules (Xiao *et al.* 2021)	Single split	Analysis of the spatial attention module	**0.868**	9.51	0.859	**9.64**	0.862	9.81
CRISPRpred	Extraction of sequence features and construction of a prediction model using SVM (Rubeis and Steger 2018)	3-fold cross-validation	Not reported	0.838	–	0.830	–	0.821	–

The best result for each column is highlighted. SPE: Spearman correlation coefficient.

In this study, to avoid information leakage, feature selection was performed independently within each training fold during cross-validation, and the selected features were subsequently applied to the corresponding test fold. To further investigate whether the performance of *DeepCC9* was influenced by the experimental conditions of the datasets used in this study (Table 1), we trained *DeepCC9* on the datasets from this study and evaluated it on independent datasets. Specifically, DeepCC9 was applied to high-quality independent datasets derived from four different cell types (HCT116, HEK293T, HeLa, and HL60), comprising a total of 15,000 sgRNAs. Its performance was then compared with that of previous methods trained on other cell types and evaluated on the same independent datasets (Hsu *et al.* 2013, Cho *et al.* 2014, Chari *et al.* 2015, Frock *et al.* 2015, Kim *et al.* 2015, Prykhozhij *et al.* 2015, Ran *et al.* 2015, Tsai *et al.* 2015, Wang *et al.* 2015, Wong *et al.* 2015, Doench *et al.* 2016, Labun *et al.* 2016). For the HeLa and HL60 datasets, DeepCC9 achieved the highest Spearman correlations (0.33 and 0.46, respectively) between the observed and predicted on-target activity values (Fig. 3G). On average, across the independent datasets, *DeepCC9* achieved a Spearman correlation of approximately 0.25, outperforming the competing methods. Overall, these results demonstrate that the performance of *DeepCC9* was not artificially inflated by the training data and support that the identified sequence features were not selected due to information leakage, despite the use of statistical techniques during the identification of the final feature subset.

### 3.2 Informative features associated with Cas9 genome-editing efficiency

**Figure 4:**
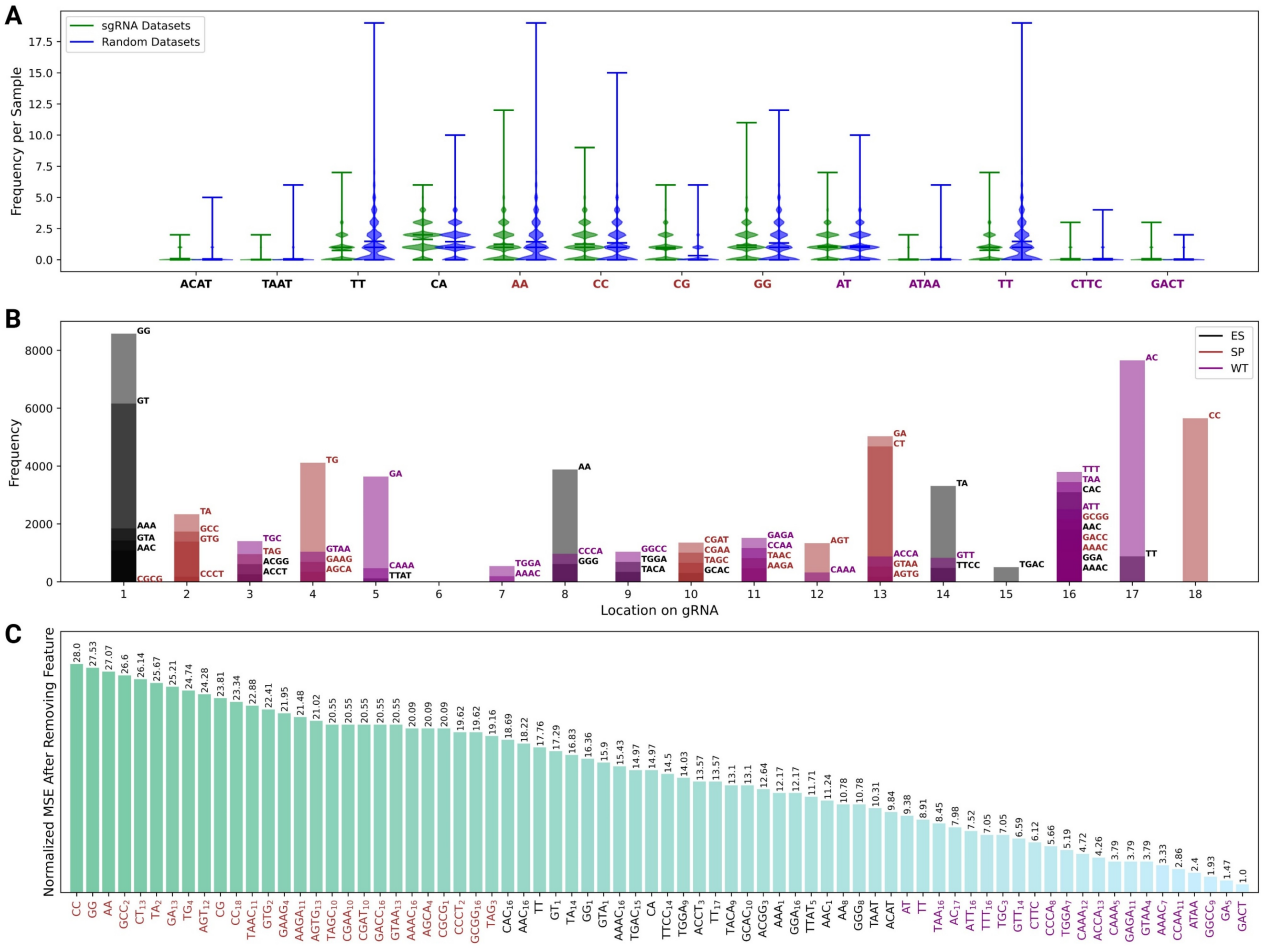
Informative features influencing Cas9 genome-editing efficiency. The numerical values following each feature indicate its presence or absence at a specific position on the sgRNA. Black, brown, and purple represent features associated with the ES, SP, and WT datasets, respectively. (A) Distribution of the total number of occurrences of each sequence motif in sgRNAs and randomly generated 20-base DNA sequences. Green and blue indicate the sgRNA and random datasets, respectively. (B) Association between the identified sequence motifs and their positions on the sgRNA. (C) Ranking of the informative features based on their impact on the SVR model. Values are normalized between 1 and 28 for better visualization. ES: eSCas9(1.1) dataset; MSE: mean squared error; SP: SpCas9-HF1 dataset; WT: WT-spCas9 dataset.

Each candidate solution in the optimization algorithms was configured to select up to 100 candidate features per run, and the final feature subset was identified based on an analysis of deep residual networks and Fisher’s exact test. For this purpose, features selected across 50 independent runs of the *DeepCC9* framework were aggregated and evaluated using Fisher’s exact test to assess their association with the sgRNA datasets and to identify those that were significantly enriched or depleted (hereafter referred to as informative features; see Tables S1–S12, available as supplementary data at *Bioinformatics* online). Because the features identified by the *Trader* algorithm enhanced the model’s ability to predict Cas9 genome-editing efficiency more effectively than those selected by the other algorithms, we primarily focus on the features derived from the SVR^*Trader*^-based *DeepCC9*. We identified 27, 25, and 22 informative features for the SP, ES, and WT datasets, respectively (Fig. 4). These features correspond to the first and second groups of extracted features described in the *Methods* section. Notably, none of the selected features were related to the PAM, indicating that the third group of features does not contribute to Cas9 genome-editing efficiency prediction.

Across all datasets, we identified 13 informative sequence features related to the total count of a sequence motif, whose distributions differed between the sgRNA and randomly generated datasets (Fig. 4A). Additionally, 61 informative features were linked to the presence or absence of a sequence motif at a specific position on the sgRNA (Fig. 4B). These findings suggest that features occurring at the first position of the sgRNA substantially influence the genome-editing efficiency of the eSpCas9(1.1) variant. Moreover, the presence of specific sequence features beginning at position 16 of the sgRNA seems to mainly affect the performance of all Cas9 variants. The enrichment or depletion of informative features was evaluated using odds ratios from Fisher’s exact test, as shown in Tables S1–S12, available as supplementary data at *Bioinformatics* online. An odds ratio greater than 1 indicates a feature is enriched in a particular Cas9 variant, while an odds ratio less than 1 indicates depletion. We also performed an ablation analysis to investigate the impact of each identified feature on model performance, as measured by the MSE of the SVR model. The features were ranked according to the increase in MSE observed after their removal, with larger increases indicating greater importance (Fig. 4C). Notably, the ranked features formed three distinct clusters associated with the datasets, likely due to several factors. First, there may be correlations among the selected features within each group, meaning removing one feature does not significantly impact the model’s performance. Second, the labels representing Cas9 genome-editing efficiency differ across the three datasets. As a result, removing features from different datasets can cause variations in their impact on performance. Third, the method may be more effective at selecting features relevant to a specific Cas9 variant than to others.

**Figure 5:**
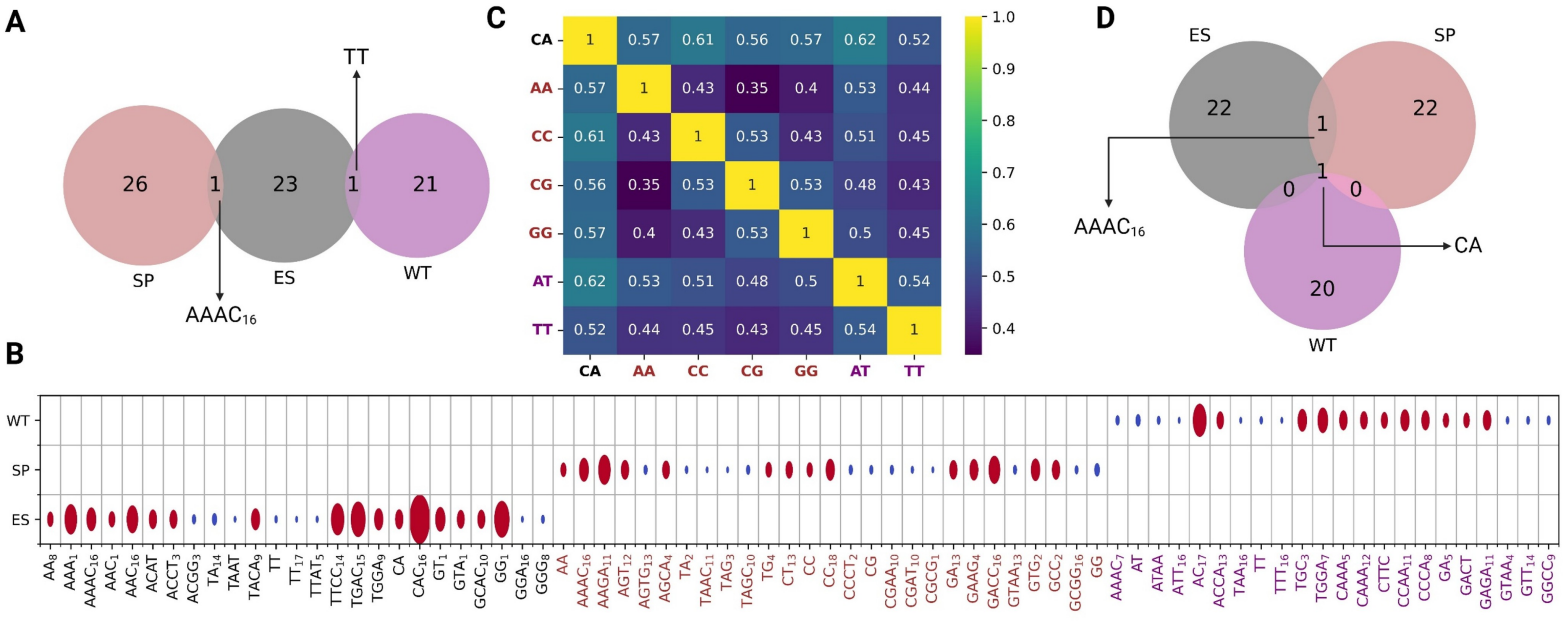
The relationship between the identified informative features. Black, brown, and purple represent features associated with the ES, SP, and WT datasets, respectively. The total number of informative features and their enrichment or depletion in each dataset are visualized in parts (A) and (B). The numerical values following each feature indicate its presence or absence at a specific position on the sgRNA. Red and blue circles denote the enrichment or depletion of a feature, respectively. Circle size also reflects the normalized odds ratio (a value between 0 and 1). The similarity scores for pairs of informative features that are moderately correlated with at least one other informative feature are shown in part (C). The total number of informative features, retained for each dataset after keeping only one feature from each group of similar features (based on pearson correlation coefficient and the cosine similarity score), is presented in part (D). ES: eSCas9(1.1) dataset; SP: SpCas9-HF1 dataset; WT: WT-spCas9 dataset.

A close comparison of features extracted from the SVR^*Trader*^-based *DeepCC9* models showed that the frequency of the TT sequence motif contributed significantly to Cas9-efficiency prediction in both the SP and ES datasets. Similarly, the sequence motif AAAC at the 16th position of the sgRNA was identified as a commonly significant feature for predictive performance of models based on the ES and WT datasets (Fig.  5A). We further examined whether various sequence motifs and position-specific features (including TT and AAAC_16_) were enriched or depleted in sgRNA sequences (Fig.  5B). We observed that the TT motif was significantly depleted in sgRNA sequences across all three datasets described above compared to randomly generated sgRNAs (odds ratio< 1). In contrast, the AAAC_16_ motif was significantly enriched in the sgRNA datasets relative to randomized sequences (odds ratio > 1). Additionally, we analyzed the relationships among all informative features within and across datasets using the Pearson correlation coefficient (PCC) and cosine similarity (CSS). We defined the similarity score between two informative features as the maximum value between their PCC and CSS values. Figure 5C shows the calculated similarity scores for pairs of informative features that exhibit moderate correlation (PCC > 0.5 or CSS > 0.5) with at least one other informative feature. Based on these scores (PCC and CSS), features were grouped, and only one representative feature from each group was retained, while the remaining redundant features were removed. As a result, the total number of informative features was reduced for each dataset (Fig.  5D). Using these reduced feature sets, we also developed CRISPR/Cas9 genome-editing efficiency prediction models for each dataset. The models achieved MSE values of 9.16, 9.22, and 9.58 across 30 independent runs for the SP, ES, and WT datasets, respectively, confirming that the grouped features (based on similarity scores) consistently contribute to the performance of *DeepCC9* and likely to the genome-editing efficiency of Cas9.

### 3.3 The association between informative features and nucleosomal DNA

**Figure 6:**
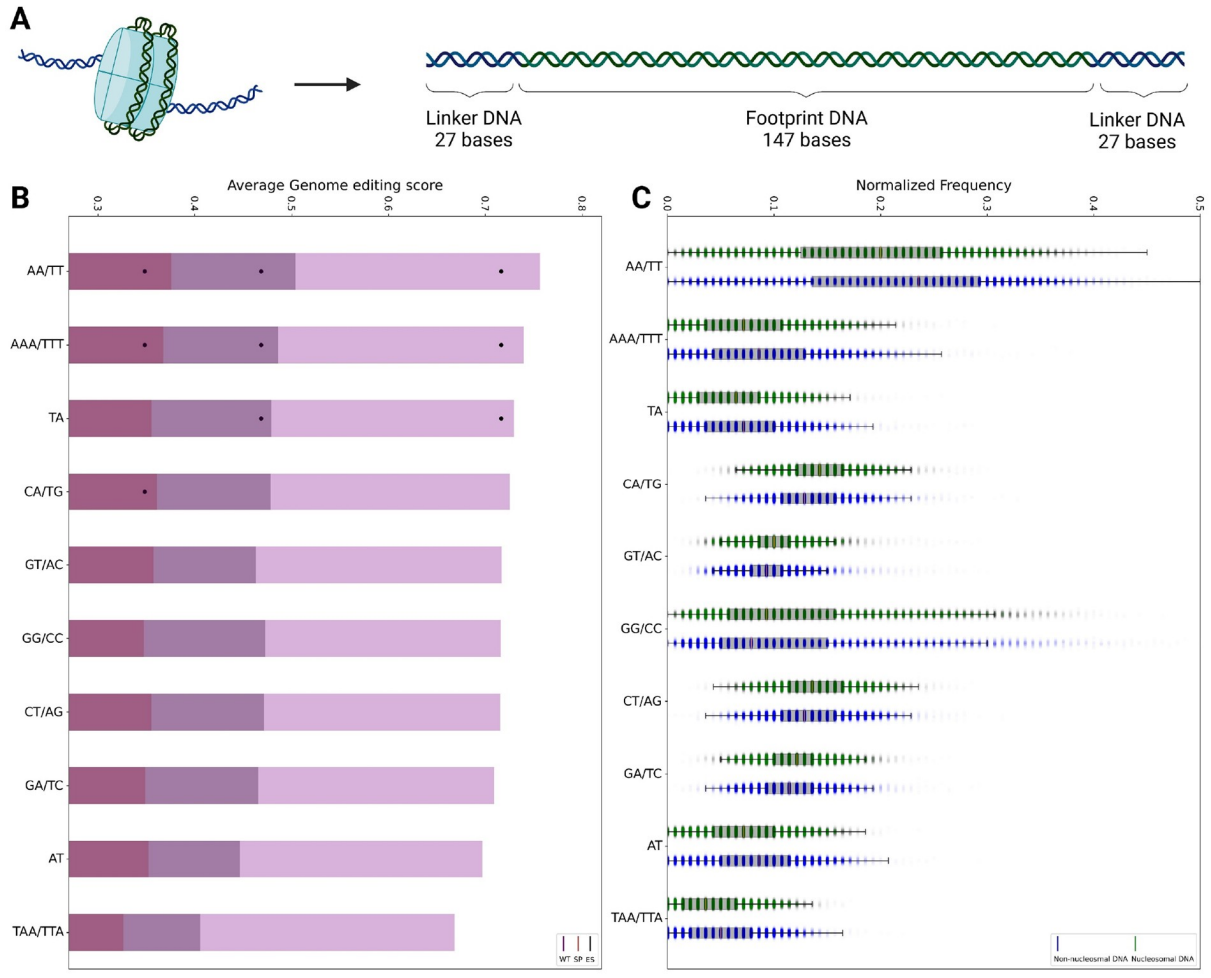
Enrichment or depletion of some sequence motifs in nucleosomal DNA. A complete list of sequence motifs is provided in Figure S1. (A) A schematic representation of a nucleosome, including histone proteins, the footprint DNA (147 bp), and the flanking linker DNA regions (27 bp). (B) Average Cas9 genome-editing scores of sgRNA sequences containing each sequence motif, illustrating how the motifs in part (C) were ranked. (C) Frequencies of specific sequence motifs in nucleosomal versus non-nucleosomal DNA. Green and blue indicate nucleosomal and non-nucleosomal DNA, respectively. Black, brown, and purple represent the ES, SP, and WT datasets, respectively. ES: eSCas9(1.1) dataset; SP: SpCas9-HF1 dataset; WT: WT-spCas9 dataset.

Previous studies have shown the essential roles of chromatin structure and DNA accessibility in Cas9 efficiency (Donovan *et al.* 2023, Park *et al.* 2023, Routhier *et al.* 2024). In particular, it has been observed that Cas9 is less efficient in modifying nucleosome-bound genomic regions. Therefore, we investigated whether there is an association between informative features and nucleosomal DNA, regardless of Cas9 category and sgRNA position. Given the symmetrical nature of a nucleosome, we also considered the complementary sequences of each informative feature in the 5′ to 3′ direction (Shaytan *et al.* 2017, Li *et al.* 2022). We performed Fisher’s exact test for both the 147-base nucleosome footprint and the 27-base flanking linker DNA regions (the target data group) (Fig. 6A, Figs S1–S2, and Tables S13–S14, available as supplementary data at *Bioinformatics* online). The control data group was selected from inter-nucleosomal (non-nucleosomal) regions that do not overlap with the target group. Each DNA sequence in both the target and control groups consists of 200 nucleotides.

We next ranked nucleosomal DNA–related sequence features based on genome-editing efficiency scores across datasets (Fig. 6B and Fig. S1B, available as supplementary data at *Bioinformatics* online). For each dataset, we analyzed sgRNA sequences containing a given motif and computed the corresponding average genome-editing score (Fig. 6B). Consistent with the trends observed in Table 1, features associated with WT Cas9 exhibited higher genome-editing rank scores than those associated with the other two Cas9 variants. Across all datasets, sgRNA sequences containing the AA motif generally displayed higher genome-editing scores than those containing other motifs, particularly CG. However, this observation alone does not imply that such motifs should be preferentially selected in sgRNA design.

In Fig. 6B, black dots represent the average Cas9 genome-editing score for each dataset and highlight motifs with scores exceeding the dataset mean. Notably, we observed a statistically significant association between the identified sequence features and nucleosomal DNA (Fig. 6C, Fig. S1, and Table S13, available as supplementary data at *Bioinformatics* online). For instance, motifs containing AC/GT and CA/TG were frequently enriched in nucleosomal DNA (*P*value < 0.01 and odds ratio > 1), whereas motifs containing AT and TA were typically depleted (*P*value < 0.01 and odds ratio < 1). A parallel analysis of the 27-bp flanking regions revealed that many motifs enriched within nucleosomal DNA were depleted in the adjacent linker regions (Fig. S2B and Table S14, available as supplementary data at *Bioinformatics* online), further supporting a chromatin-dependent bias in the distribution of these sequence features. The enrichment or depletion of sequence motifs in nucleosomal DNA was determined using the odds ratio from Fisher’s exact test. Specifically, an odds ratio greater than 1 and a *P*value < 0.01 indicate enrichment of a sequence motif in nucleosomal DNA compared with non-nucleosomal regions, while an odds ratio less than 1 and a *P*value < 0.01 indicate depletion. We note that although some odds ratios are close to 1, this is not unexpected given the very large number of sequences analyzed. In such large datasets, even modest deviations from 1 may reflect consistent, reproducible sequence preferences rather than noise. To assess the robustness of these enrichment or depletion patterns, we also calculated 95% confidence intervals for all odds ratios (Tables S13–S14, available as supplementary data at *Bioinformatics* online). These intervals quantify the uncertainty of the estimates and indicate whether the observed effects are statistically supported.

Nucleosome positioning is not the sole factor affecting the genome-editing efficiency of Cas9. Therefore, we investigated whether DNA methylation could also influence Cas9 genome-editing efficiency. Since the sgRNA datasets used in this study were derived from the HEK293T cell line, we obtained the genomic coordinates of CpG methylation sites from the ENCODE Project Consortium and mapped them to the genomic coordinates of sgRNAs containing CpG sites. For each Cas9 variant, the sgRNAs were divided into two groups: (i) sgRNAs with genome-editing scores higher than the average genome-editing score, and (ii) sgRNAs with genome-editing scores lower than the average. Fisher’s exact test was then performed to evaluate the association between methylation at sgRNA binding sites and genome-editing efficiency (Table S15, available as supplementary data at *Bioinformatics* online). The results showed that, for all Cas9 variants, methylated sgRNA binding sites were associated with higher genome-editing efficiency compared with unmethylated sites (*P* value < 0.01 and odds ratio ≈ 1.22). These findings suggest that DNA methylation may represent an important factor influencing Cas9 activity and could be considered in future sgRNA design strategies.

## 4 Discussion

Over the last decade, CRISPR-Cas9-based genome editing has become a key technique with numerous applications. Despite widespread adoption, achieving optimal efficiency while avoiding off-target effects remains a significant challenge for CRISPR/Cas9 experiments. Because Cas9 genome-editing efficiency depends on the sgRNA sequence, this work develops an interpretable deep learning framework, *DeepCC9*, to predict genome-editing efficiency. Our method systematically searches the sequence-based feature space to identify sequence motifs, along with their frequencies and positions on the sgRNA, and uses these to train the *DeepCC9* model. We applied *DeepCC9* to datasets related to three Cas9 variants, achieving higher interpretability and lower MSE than previous methods for predicting Cas9 editing efficiency (Table 2). Additionally, *DeepCC9* can identify di-, tri-, and tetra-nucleotide sequence motifs, whereas earlier studies have analyzed their models at single-nucleotide resolution. The improved predictive performance of *DeepCC9* stems from a systematic evaluation of widely used and effective metaheuristic algorithms for constructing the candidate feature set; notably, the *Trader* optimization algorithm outperformed the other methods, leading to faster convergence, greater stability, and lower MSE in the optimized *DeepCC9* model (Figs 2 and 3).

Overall, we identified 74 informative features that influence the prediction of Cas9 genome-editing efficiency (Figs 4 and 5). These features are either enriched or depleted in sgRNA sequences (Tables S1–S12, available as supplementary data at *Bioinformatics* online). Interestingly, our results show that the efficiency of different Cas9 variants depends on specific positions within an sgRNA. For example, eSpCas9(1.1)-related features were found at the first position of the sgRNA, while wild-type WT-SpCas9-related features appeared at positions 16 and 17 (Fig. 4). There may be several reasons underlying the contribution of these position-specific sequence features to Cas9 genome-editing efficiency. First, sequence motifs near the 5′ region of the sgRNA may affect its folding and stability, thereby influencing Cas9 loading and guide RNA functionality (e.g. AA or GG at the first position of the 5′ region). In addition, other motifs located at early positions within the sgRNA sequence (e.g. ACCT or ACGG at the third position of the 5′ region) may influence sgRNA secondary structure formation and accessibility during Cas9 complex assembly. Second, motifs located near the 3′ region of the sgRNA, corresponding to the PAM-proximal region (e.g. AAC, CAC, or GGA at the 16th position of the 5′ region), may strongly influence target recognition and cleavage efficiency by altering Cas9 binding strength and mismatch sensitivity (Zhang *et al.* 2015). Furthermore, some motifs identified near the PAM-proximal region (e.g. TA at the 14th position of the 5′ region) may directly affect DNA–RNA hybridization stability and consequently influence Cas9 genome-editing efficiency (Corsi *et al.* 2022). In addition to early- and late-position motifs, several motifs located within the central region of the sgRNA (e.g. TACA and TGGA at the ninth position, and GCAC at the tenth position) may contribute to local thermodynamic properties and RNA folding patterns that affect Cas9 genome-editing activity (Jung *et al.* 2024).

In addition to position-specific motifs, several composition-based sequence features may also contribute to Cas9 genome-editing efficiency. For example, dinucleotide motifs such as TT and CA may reflect global nucleotide composition biases associated with guide RNA stability and Cas9 activity. Similarly, composition-based 4-mer motifs, including TAAT and ACAT, may capture sequence patterns related to DNA accessibility, structural flexibility, or local conformational preferences that influence Cas9-mediated genome editing. We also used Fisher’s exact test to identify enrichment or depletion of sequence features within sgRNAs (Tables S1–S3, available as supplementary data at *Bioinformatics* online), revealing that the TT/TTT motif is significantly depleted. This observation is consistent with previous studies reporting depletion of A/T-rich motifs in highly active sgRNAs, potentially because A–T base pairs form only two hydrogen bonds, resulting in weaker sgRNA–target DNA hybridization compared with G–C base pairs, which form three hydrogen bonds(Jung *et al.* 2024). We also observed differences in the enrichment and depletion patterns of several sequence features among datasets derived from different Cas9 variants (Tables S1–S3, available as supplementary data at *Bioinformatics* online).

The enrichment or depletion of specific motifs is likely influenced by additional factors, including position-specific sequence context and nucleosome occupancy. These factors can render certain motifs more favorable or unfavorable for sgRNA binding or cleavage, explaining why only specific motif patterns, rather than all A/T- or G/C-rich motifs, show significant enrichment or depletion. For this reason, we also paid attention to position-specific features in our analysis. Our position-specific sequence analyses identified ten sequence motifs starting at the 16th position of the sgRNA that appear associated with Cas9 efficiency. For example, in WT-SpCas9, the TTT sequence motif was significantly depleted, likely because it forms a less stable binding interface between the sgRNA and the target DNA. In contrast, the CC motif in SpCas9-HF1 variant, starting at position 18, is enriched in efficient sgRNAs because it contributes to strong GC base pairing at a critical position near the PAM. As a result, CC may promote stronger, more stable binding, greater specificity, and higher Cas9 cleavage efficiency (Xu *et al.* 2015, Doench *et al.* 2016, Haeussler *et al.* 2016).

Finally, we examined the association between the identified sequence motifs and the underlying nucleosome occupancy profiles (Fig. 6), as nucleosomes regulate DNA accessibility and can influence Cas9 efficiency (Isaac *et al.* 2016, Chen *et al.* 2017, Handelmann *et al.* 2023). Interestingly, we observed that most sequence motifs identified by our model were significantly enriched or depleted in nucleosomal regions, underscoring the role of nucleosomes in Cas9 genome-editing efficiency. Previous studies have shown that nucleosomes inhibit DNA accessibility to the Cas9 enzyme in human and mouse genomes (Horlbeck *et al.* 2016). Therefore, in addition to weak binding between the sgRNA and the target DNA, nucleosome positioning can also inhibit Cas9 efficiency. However, our approach has limitations because it primarily relies on enrichment or depletion patterns for sequence motifs, without considering their structural-functional relationships in nucleosomes. For instance, our sequence analyses showed depletion of the TT dinucleotide among Cas9 sgRNAs; one would expect this motif to be enriched in nucleosomes (supporting the idea that lower Cas9 efficiency is due to reduced DNA accessibility), but this is not the case. Previous studies have shown that the low association of the TT dinucleotide with nucleosomes stems from the known structural preference of AA/TT dinucleotides for minor-groove sites facing inward toward the histone octamer, making them less accessible to Cas9 binding. In contrast, C/G nucleotides tend to be enriched at minor groove sites that face outward from the histone octamer and are typically more accessible (Masoudi-Sobhanzadeh *et al.* 2024). Similarly, comparing the enrichment or depletion of sequence motifs in nucleosomal DNA and in the flanking linker DNA revealed opposite trends (Fig. 6 and Figs S1–S2, available as supplementary data at *Bioinformatics* online). For example, AA/TT dinucleotides are depleted in nucleosomal regions but enriched in flanking linker DNA. Therefore, when designing an sgRNA, it may be important to consider whether the target DNA resides within a nucleosome and whether its minor groove sites face the histone core. Therefore, we anticipate that future work to improve the predictive performance of Cas9 efficiency prediction should include both sequence and structural features. Despite these limitations, our work offers a highly interpretable deep learning framework that achieves better performance in predicting Cas9 efficiency across all three distinct Cas9 variants compared to earlier methods.

## Supplementary Material

btag483_Supplementary_Data

## Data Availability

The data used in this study were obtained from previously published studies and have been made publicly available in our repository at https://zenodo.org/records/20073890.
