# Supplementary material for: An interpretable deep learning framework uncovers features governing CRISPR-Cas9 genome-editing efficiency
Source: Bioinformatics. 2026 Jul 2;42(7):btag483. doi: 10.1093/bioinformatics/btag483 (PMC13384063; doi:10.1093/bioinformatics/btag483)
Supplement: btag483_Supplementary_Data [file btag483_supplementary_data.docx]

**An interpretable deep learning framework uncovers features governing CRISPR‑Cas9 genome‑editing efficiency**

Nasim Bakhtiyari^1,2^, Yosef Masoudi-Sobhanzadeh^*3,4^, Safar Farajnia^*1,4^, and Sushant Kumar^*2,5^

^1^Drug Applied Research Center, Tabriz University of Medical Sciences, Tabriz, Iran

^2^Department of Computer Engineering, Istanbul Rumeli University, Istanbul, Turkey

^3^Faculty of Advanced Medical Sciences, Tabriz University of Medical Sciences, Tabriz, Iran

^4^Princess Margaret Cancer Centre, University Health Network, Toronto, Ontario, Canada.

^5^Department of Medical Biophysics, University of Toronto, Toronto, Canada

# First author:

Nasim Bakhtiyari

Email: bakhtiyari.nasim1@gmail.com

* Corresponding authors:

Yosef Masoudi-Sobhanzadeh

Email: [yosef.masoudi-sobhanzadeh@uhn.ca](mailto:yosef.masoudi-sobhanzadeh@uhn.ca)

Safar Farajnia

Email: [farajnia@gmail.com](mailto:farajnia@gmail.com)

Sushant Kumar

Email: [sushant.kumar@uhn.ca](mailto:sushant.kumar@uhn.ca)

**Table S1.** Features identified by SVR*^Trader^-*based *DeepCC9* on the eSpCas9 (1.1) (ES) dataset. The numerical values following each feature indicate its presence or absence at a specific position on the sgRNA.

| Feature | Elements of contingency table | | | | P-value | Odds ratio |
| --- | --- | --- | --- | --- | --- | --- |
|  | a | b | c | d |  |  |
| AA_8 | 3879 | 53009 | 3513 | 53375 | 1.12757E-05 | 1.111808286 |
| AAA_1 | 1844 | 55044 | 854 | 56034 | 1.30292E-84 | 2.198086028 |
| AAAC_16 | 389 | 56499 | 227 | 56661 | 6.09529E-11 | 1.718569967 |
| AAC_1 | 1077 | 55811 | 921 | 55967 | 0.000465118 | 1.172649701 |
| AAC_16 | 1804 | 55084 | 907 | 55981 | 3.63465E-69 | 2.021363543 |
| ACAT | 5429 | 279011 | 3836 | 280604 | 9.95134E-63 | 1.423356782 |
| ACCT_3 | 258 | 56630 | 188 | 56700 | 0.001039487 | 1.374036767 |
| ACGG_3 | 161 | 56727 | 216 | 56672 | 0.005272009 | 0.744647692 |
| TA_14 | 3310 | 53578 | 3630 | 53258 | 7.74188E-05 | 0.90639964 |
| TAAT | 1808 | 282632 | 3862 | 280578 | 4.9598E-169 | 0.464748974 |
| TACA_9 | 339 | 56549 | 214 | 56674 | 1.11124E-07 | 1.587613786 |
| TT | 42636 | 526244 | 67562 | 501318 | 0 | 0.601173872 |
| TT_17 | 879 | 56009 | 3587 | 53301 | 0 | 0.233203485 |
| TTAT_5 | 118 | 56770 | 219 | 56669 | 3.90073E-08 | 0.537854179 |
| TTCC_14 | 477 | 56411 | 206 | 56682 | 8.32933E-26 | 2.32665787 |
| TGAC_15 | 507 | 56381 | 198 | 56690 | 2.64973E-32 | 2.574639641 |
| TGGA_9 | 315 | 56573 | 197 | 56691 | 1.94598E-07 | 1.602319935 |
| CA | 92868 | 476012 | 67633 | 501247 | 0 | 1.445910215 |
| CAC_16 | 3095 | 53793 | 899 | 55989 | 5.973E-289 | 3.583256581 |
| GT_1 | 6161 | 50727 | 3619 | 53269 | 9.3725E-161 | 1.787713793 |
| GTA_1 | 1224 | 55664 | 900 | 55988 | 1.3599E-12 | 1.367916068 |
| GCAC_10 | 305 | 56583 | 221 | 56667 | 0.00027868 | 1.382139304 |
| GG_1 | 8575 | 48313 | 3550 | 53338 | 0 | 2.666726624 |
| GGA_16 | 450 | 56438 | 884 | 56004 | 2.40624E-33 | 0.505135255 |
| GGG_8 | 614 | 56274 | 920 | 55968 | 3.66837E-15 | 0.663762244 |

Contingency table used in Fisher’s exact test to assess the association between the presence or absence of a specific sequence motif (informative features) and the sgRNA. An odds ratio greater than 1 indicates that the feature is enriched in the sgRNA dataset, whereas an odds ratio less than 1 indicates it is depleted.

Odds Ratio = (a*d)/(c*b)

|  | a given sequence motif | other sequence motifs |
| --- | --- | --- |
| sgRNA dataset | a | b |
| Random DNA sequence of 20 bases dataset | c | d |

**Table S2.** Features identified by SVR*^Trader^-*based *DeepCC9* on the SpCas9-HF1 (SP) dataset. The numerical values following each feature indicate its presence or absence at a specific position on the sgRNA.

| Feature | Elements of contingency table | | | | P-value | Odds ratio |
| --- | --- | --- | --- | --- | --- | --- |
|  | a | b | c | d |  |  |
| AA | 72825 | 513348 | 69662 | 516508 | 3.97144E-19 | 1.051840121 |
| AAAC_16 | 400 | 58217 | 233 | 58384 | 2.86031E-11 | 1.721662795 |
| AAGA_11 | 465 | 58152 | 215 | 58402 | 3.41731E-22 | 2.172088704 |
| AGT_12 | 1333 | 57284 | 939 | 57678 | 7.04259E-17 | 1.429359307 |
| AGTG_13 | 174 | 58443 | 234 | 58383 | 0.003383598 | 0.742826344 |
| AGCA_4 | 339 | 58278 | 253 | 58364 | 0.000451706 | 1.341898251 |
| TA_2 | 2337 | 56280 | 3686 | 54931 | 1.07403E-71 | 0.6188235 |
| TAAC_11 | 123 | 58494 | 252 | 58365 | 2.2951E-11 | 0.487018815 |
| TAG_3 | 431 | 58186 | 941 | 57676 | 1.87126E-44 | 0.454008807 |
| TAGC_10 | 168 | 58449 | 228 | 58389 | 0.002932038 | 0.73608571 |
| TG_4 | 4112 | 54505 | 3651 | 54966 | 6.50818E-08 | 1.135792673 |
| CT_13 | 4679 | 53938 | 3737 | 54880 | 1.61885E-26 | 1.273940695 |
| CC | 74531 | 511639 | 69234 | 516936 | 2.70141E-50 | 1.087653749 |
| CC_18 | 5651 | 52966 | 3684 | 54933 | 1.6175E-100 | 1.590896136 |
| CCCT_2 | 175 | 58442 | 231 | 58386 | 0.006181858 | 0.756849837 |
| CG | 50921 | 535249 | 69385 | 516785 | 0 | 0.708574251 |
| CGAA_10 | 161 | 58456 | 218 | 58399 | 0.003903966 | 0.737811973 |
| CGAT_10 | 145 | 58472 | 248 | 58369 | 2.19151E-07 | 0.583647494 |
| GA_13 | 4924 | 53693 | 3572 | 55045 | 1.67588E-52 | 1.413210319 |
| GAAG_4 | 390 | 58227 | 251 | 58366 | 4.10497E-08 | 1.557494069 |
| GACC_16 | 454 | 58163 | 224 | 58393 | 5.28489E-19 | 2.034800444 |
| CGCG_1 | 5 | 58612 | 193 | 58424 | 1.359E-49 | 0.0980041 |
| GTAA_13 | 177 | 58440 | 246 | 58371 | 0.000904135 | 0.718662668 |
| GTG_2 | 1387 | 57230 | 858 | 57759 | 1.26281E-29 | 1.631492542 |
| GCC_2 | 1329 | 57288 | 929 | 57688 | 1.88979E-17 | 1.440559128 |
| GCGG_16 | 165 | 58452 | 246 | 58371 | 7.34428E-05 | 0.669802239 |
| GG | 68517 | 517654 | 69697 | 516474 | 0.00073387 | 0.980828651 |

Contingency table used in Fisher’s exact test to assess the association between the presence or absence of a specific sequence motif (informative features) and the sgRNA. An odds ratio greater than 1 indicates that the feature is enriched in the sgRNA dataset, whereas an odds ratio less than 1 indicates it is depleted.

Odds Ratio = (a*d)/(c*b)

|  | a given sequence motif | other sequence motifs |
| --- | --- | --- |
| sgRNA dataset | a | b |
| Random DNA sequence of 20 bases dataset | c | d |

**Table S3.** Features identified by SVR*^Trader^-*based *DeepCC9* on the WT-SpCas9 (WT) dataset. The numerical values following each feature indicate its presence or absence at a specific position on the sgRNA.

| Feature | Elements of contingency table | | | | P-value | Odds ratio |
| --- | --- | --- | --- | --- | --- | --- |
|  | a | b | c | d |  |  |
| AAAC_7 | 193 | 55411 | 257 | 55347 | 0.002880604 | 0.750105385 |
| AT | 60722 | 495318 | 66155 | 489885 | 4.69671E-59 | 0.907806786 |
| ATAA | 2491 | 275529 | 3748 | 274272 | 6.17269E-58 | 0.661589041 |
| ATT_16 | 277 | 55327 | 887 | 54717 | 1.45414E-75 | 0.308845519 |
| AC_17 | 7650 | 47954 | 3465 | 52139 | 0 | 2.400468739 |
| ACCA_13 | 298 | 55306 | 230 | 55374 | 0.003431155 | 1.297245208 |
| TAA_16 | 441 | 55163 | 856 | 54748 | 1.9645E-31 | 0.511311083 |
| TT | 41871 | 514169 | 66045 | 489995 | 0 | 0.604169988 |
| TTT_16 | 131 | 55473 | 866 | 54738 | 1.586E-134 | 0.149265925 |
| TGC_3 | 1405 | 54199 | 860 | 54744 | 3.94021E-31 | 1.65014887 |
| TGGA_7 | 437 | 55167 | 237 | 55367 | 9.56578E-15 | 1.850566584 |
| CAAA_5 | 280 | 55324 | 194 | 55410 | 8.76715E-05 | 1.445542547 |
| CAAA_12 | 322 | 55282 | 242 | 55362 | 0.000837965 | 1.332504027 |
| CTTC | 4377 | 273643 | 3612 | 274408 | 6.92526E-18 | 1.215181727 |
| CCAA_11 | 340 | 55264 | 214 | 55390 | 8.98646E-08 | 1.592407421 |
| CCCA_8 | 283 | 55321 | 196 | 55408 | 7.88397E-05 | 1.44614825 |
| GA_5 | 3638 | 51966 | 3376 | 52228 | 0.001282209 | 1.083039667 |
| GACT | 4123 | 273897 | 3676 | 274344 | 3.63026E-07 | 1.123430016 |
| GAGA_11 | 388 | 55216 | 263 | 55341 | 1.01632E-06 | 1.478624976 |
| GTAA_4 | 131 | 55473 | 215 | 55389 | 7.12919E-06 | 0.608379689 |
| GTT_14 | 572 | 55032 | 882 | 54722 | 2.66909E-16 | 0.644872874 |
| GGCC_9 | 152 | 55452 | 211 | 55393 | 0.002250497 | 0.719612676 |

Contingency table used in Fisher’s exact test to assess the association between the presence or absence of a specific sequence motif (informative features) and the sgRNA. An odds ratio greater than 1 indicates that the feature is enriched in the sgRNA dataset, whereas an odds ratio less than 1 indicates it is depleted.

Odds Ratio = (a*d)/(c*b)

|  | a given sequence motif | other sequence motifs |
| --- | --- | --- |
| sgRNA dataset | a | b |
| Random DNA sequence of 20 bases dataset | c | d |

**Table S4.** Features identified by SVR*^WCC^-*based *DeepCC9* on the eSpCas9 (1.1) (ES) dataset. The numerical values following each feature indicate its presence or absence at a specific position on the sgRNA.

| Feature | Elements of contingency table | | | | P-value | Odds ratio |
| --- | --- | --- | --- | --- | --- | --- |
|  | a | b | c | d |  |  |
| AA | 70793 | 498090 | 67504 | 501376 | 3.9564E-21 | 1.055641676 |
| AA_1 | 5944 | 50944 | 3643 | 53245 | 3.0829E-134 | 1.705318169 |
| AAT_4 | 632 | 56256 | 897 | 55991 | 9.5051E-12 | 0.701251834 |
| AACA_2 | 284 | 56604 | 203 | 56685 | 0.000272086 | 1.40101676 |
| ATT_1 | 1208 | 55680 | 915 | 55973 | 1.48371E-10 | 1.32716585 |
| ATGG | 4159 | 280281 | 3860 | 280580 | 0.000802753 | 1.078610561 |
| AC_17 | 7937 | 48951 | 3545 | 53343 | 0 | 2.439810012 |
| ACAT_6 | 298 | 56590 | 212 | 56676 | 0.000156519 | 1.407796564 |
| ACC_14 | 1436 | 55452 | 843 | 56045 | 2.18757E-36 | 1.72165657 |
| AG_14 | 3756 | 53132 | 3524 | 53364 | 0.005133091 | 1.070488227 |
| TT_11 | 2449 | 54439 | 3525 | 53363 | 1.52007E-46 | 0.681019836 |
| TTAC_11 | 164 | 56724 | 239 | 56649 | 0.00021353 | 0.685285191 |
| TCT_12 | 1104 | 55784 | 895 | 55993 | 2.63253E-06 | 1.23814105 |
| TGAA_9 | 366 | 56522 | 212 | 56676 | 1.38721E-10 | 1.73111889 |
| TGCA_4 | 294 | 56594 | 223 | 56665 | 0.002002629 | 1.320039631 |
| TGG_15 | 518 | 56370 | 881 | 56007 | 1.20902E-22 | 0.58418194 |
| CATC_13 | 548 | 56340 | 221 | 56667 | 4.39327E-33 | 2.494029944 |
| CTC_16 | 1405 | 55483 | 918 | 55970 | 1.53728E-24 | 1.543935007 |
| CCGA_3 | 151 | 56737 | 219 | 56669 | 0.000469345 | 0.688671345 |
| GAT_12 | 1001 | 55887 | 867 | 56021 | 0.001909315 | 1.157324213 |
| GATT | 2658 | 281782 | 3794 | 280646 | 4.64685E-46 | 0.697755485 |
| GAC_16 | 1590 | 55298 | 819 | 56069 | 1.41447E-57 | 1.968460067 |
| GACA_11 | 426 | 56462 | 187 | 56701 | 1.59309E-22 | 2.28771781 |
| GAGA_1 | 609 | 56279 | 219 | 56669 | 1.53166E-43 | 2.800092348 |
| GTGG_11 | 151 | 56737 | 212 | 56676 | 0.001573789 | 0.71149837 |
| GCGT_16 | 108 | 56780 | 228 | 56660 | 4.9996E-11 | 0.472683117 |
| GGAC_14 | 176 | 56712 | 246 | 56642 | 0.000746272 | 0.714564073 |
| GGGC_15 | 88 | 56800 | 227 | 56661 | 2.41881E-15 | 0.386716511 |

Contingency table used in Fisher’s exact test to assess the association between the presence or absence of a specific sequence motif (informative features) and the sgRNA. An odds ratio greater than 1 indicates that the feature is enriched in the sgRNA dataset, whereas an odds ratio less than 1 indicates it is depleted.

Odds Ratio = (a*d)/(c*b)

|  | a given sequence motif | other sequence motifs |
| --- | --- | --- |
| sgRNA dataset | a | b |
| Random DNA sequence of 20 bases dataset | c | d |

**Table S5.** Features identified by SVR*^WCC^-*based *DeepCC9* on the SpCas9-HF1 (SP) dataset. The numerical values following each feature indicate its presence or absence at a specific position on the sgRNA.

| Feature | Elements of contingency table | | | | P-value | Odds ratio |
| --- | --- | --- | --- | --- | --- | --- |
|  | a | b | c | d |  |  |
| AA | 72825 | 513348 | 69662 | 516508 | 3.97144E-19 | 1.051840121 |
| AC_12 | 5124 | 53493 | 3706 | 54911 | 1.2317E-55 | 1.419273534 |
| ACGT_12 | 184 | 58433 | 248 | 58369 | 0.002353978 | 0.741122863 |
| TA_2 | 2337 | 56280 | 3686 | 54931 | 1.07403E-71 | 0.6188235 |
| TAT_17 | 260 | 58357 | 931 | 57686 | 8.83593E-90 | 0.276058507 |
| TACC_16 | 388 | 58229 | 219 | 58398 | 5.97096E-12 | 1.776831532 |
| TT | 43736 | 542434 | 69584 | 516586 | 0 | 0.598584407 |
| TTA_3 | 592 | 58025 | 911 | 57706 | 1.17054E-16 | 0.646262791 |
| TTA_17 | 83 | 58534 | 929 | 57688 | 4.9587E-183 | 0.088052088 |
| TTGA_12 | 136 | 58481 | 224 | 58393 | 3.94905E-06 | 0.606229252 |
| CAT | 18653 | 391666 | 16620 | 393699 | 1.90523E-28 | 1.128148083 |
| CTC_9 | 1150 | 57467 | 925 | 57692 | 6.83114E-07 | 1.248110902 |
| CCCA_16 | 525 | 58092 | 229 | 58388 | 9.09704E-28 | 2.304257935 |
| CG_18 | 4696 | 53921 | 3629 | 54988 | 6.72622E-34 | 1.319626737 |
| CGA | 12250 | 398069 | 16419 | 393900 | 4.8905E-139 | 0.738273039 |
| CGA_13 | 601 | 58016 | 943 | 57674 | 1.76858E-18 | 0.633570678 |
| GACG_12 | 110 | 58507 | 240 | 58377 | 2.82865E-12 | 0.457314937 |
| GTAT_8 | 141 | 58476 | 202 | 58415 | 0.001145987 | 0.697291654 |
| GTGC_6 | 265 | 58352 | 195 | 58422 | 0.001242426 | 1.360604607 |
| GCCA_14 | 400 | 58217 | 216 | 58401 | 9.50584E-14 | 1.857704794 |
| GGAA | 4777 | 288308 | 3940 | 289145 | 1.74522E-19 | 1.215956428 |
| GGTA_10 | 134 | 58483 | 211 | 58406 | 3.89614E-05 | 0.634234942 |
| GGGC_10 | 149 | 58468 | 226 | 58391 | 8.02512E-05 | 0.658423774 |
| GGGG_9 | 90 | 58527 | 238 | 58379 | 1.31164E-16 | 0.377195011 |

Contingency table used in Fisher’s exact test to assess the association between the presence or absence of a specific sequence motif (informative features) and the sgRNA. An odds ratio greater than 1 indicates that the feature is enriched in the sgRNA dataset, whereas an odds ratio less than 1 indicates it is depleted.

Odds Ratio = (a*d)/(c*b)

|  | a given sequence motif | other sequence motifs |
| --- | --- | --- |
| sgRNA dataset | a | b |
| Random DNA sequence of 20 bases dataset | c | d |

**Table S6.** Features identified by SVR*^WCC^-*based *DeepCC9* on the WT-SpCas9 (WT) dataset. The numerical values following each feature indicate its presence or absence at a specific position on the sgRNA.

| Feature | Elements of contingency table | | | | P-value | Odds ratio |
| --- | --- | --- | --- | --- | --- | --- |
|  | a | b | c | d |  |  |
| AA | 68856 | 487187 | 66037 | 490003 | 2.71446E-16 | 1.048715054 |
| AAAC_6 | 149 | 55455 | 213 | 55391 | 0.000886479 | 0.698723196 |
| AAAG_1 | 568 | 55036 | 216 | 55388 | 1.55399E-37 | 2.646448251 |
| ATTT_1 | 310 | 55294 | 229 | 55375 | 0.000540069 | 1.355694838 |
| TA_16 | 2824 | 52780 | 3568 | 52036 | 9.4647E-22 | 0.780322924 |
| TAA_11 | 509 | 55095 | 908 | 54696 | 8.99029E-27 | 0.556512999 |
| TAAT_5 | 100 | 55504 | 223 | 55381 | 5.92817E-12 | 0.447436746 |
| TT | 41871 | 514169 | 66045 | 489995 | 0 | 0.604169988 |
| TCG_4 | 496 | 55108 | 933 | 54671 | 1.27583E-31 | 0.527402763 |
| TGTT | 2681 | 275339 | 3696 | 274324 | 1.68308E-37 | 0.722704777 |
| CTCC_14 | 418 | 55186 | 221 | 55383 | 4.5869E-15 | 1.898154542 |
| CCCC_13 | 280 | 55324 | 220 | 55384 | 0.008117515 | 1.274107571 |
| CCCG_11 | 124 | 55480 | 225 | 55379 | 6.75071E-08 | 0.550107827 |
| CGCT | 2386 | 275634 | 3715 | 274305 | 4.96943E-66 | 0.63916437 |
| CGGG_11 | 86 | 55518 | 226 | 55378 | 1.07065E-15 | 0.379571387 |
| GATA_3 | 133 | 55471 | 244 | 55360 | 1.09676E-08 | 0.543991233 |
| GTAA_4 | 131 | 55473 | 215 | 55389 | 7.12919E-06 | 0.608379689 |
| GTAA_7 | 141 | 55463 | 222 | 55382 | 2.42062E-05 | 0.634207563 |
| GGAC_3 | 297 | 55307 | 189 | 55415 | 1.04308E-06 | 1.574497157 |
| GGAG | 4988 | 273032 | 3622 | 274398 | 6.30747E-50 | 1.384029637 |
| GGTT | 2143 | 275877 | 3753 | 274267 | 9.7258E-100 | 0.567677483 |

Contingency table used in Fisher’s exact test to assess the association between the presence or absence of a specific sequence motif (informative features) and the sgRNA. An odds ratio greater than 1 indicates that the feature is enriched in the sgRNA dataset, whereas an odds ratio less than 1 indicates it is depleted.

Odds Ratio = (a*d)/(c*b)

|  | a given sequence motif | other sequence motifs |
| --- | --- | --- |
| sgRNA dataset | a | b |
| Random DNA sequence of 20 bases dataset | c | d |

**Table S7.** Features identified by SVR*^WAA^-*based *DeepCC9* on the eSpCas9 (1.1) (ES) dataset. The numerical values following each feature indicate its presence or absence at a specific position on the sgRNA.

| Feature | Elements of contingency table | | | | P-value | Odds ratio |
| --- | --- | --- | --- | --- | --- | --- |
|  | a | b | c | d |  |  |
| AA | 70793 | 498090 | 67504 | 501376 | 3.9564E-21 | 1.055641676 |
| AA_1 | 5944 | 50944 | 3643 | 53245 | 3.0829E-134 | 1.705318169 |
| AA_10 | 3861 | 53027 | 3505 | 53383 | 1.88745E-05 | 1.108964639 |
| AA_16 | 4124 | 52764 | 3618 | 53270 | 2.72889E-09 | 1.15078735 |
| AAA_1 | 1844 | 55044 | 854 | 56034 | 1.30292E-84 | 2.198086028 |
| AAA_4 | 779 | 56109 | 897 | 55991 | 0.003974081 | 0.866623996 |
| AAA_8 | 1102 | 55786 | 823 | 56065 | 1.54128E-10 | 1.345700344 |
| AAA_10 | 1196 | 55692 | 881 | 56007 | 3.24974E-12 | 1.365226681 |
| AAA_11 | 1157 | 55731 | 940 | 55948 | 1.88928E-06 | 1.235643633 |
| AAA_14 | 1066 | 55822 | 849 | 56039 | 6.26034E-07 | 1.260475762 |
| AAGA_9 | 440 | 56448 | 216 | 56672 | 1.16466E-18 | 2.045120517 |
| AC_15 | 4849 | 52039 | 3629 | 53259 | 3.19431E-43 | 1.367506128 |
| AGTT_15 | 65 | 56823 | 214 | 56674 | 8.97654E-20 | 0.302941862 |
| AGGT_13 | 122 | 56766 | 221 | 56667 | 9.54254E-08 | 0.551073447 |
| AGGG_13 | 88 | 56800 | 194 | 56694 | 2.38082E-10 | 0.452761725 |
| TAT_17 | 252 | 56636 | 895 | 55993 | 1.272E-85 | 0.27836759 |
| TATA_9 | 174 | 56714 | 229 | 56659 | 0.006971674 | 0.759088465 |
| TTT | 6396 | 391820 | 15962 | 382255 | 0 | 0.39091985 |
| TTC_14 | 1652 | 55236 | 916 | 55972 | 2.25309E-49 | 1.827524357 |
| TCTA_8 | 146 | 56742 | 246 | 56642 | 4.77918E-07 | 0.59244998 |
| TGA_9 | 1205 | 55683 | 864 | 56024 | 4.0237E-14 | 1.403216854 |
| CA_18 | 6536 | 50352 | 3543 | 53345 | 7.1274E-217 | 1.954419941 |
| CTAC_13 | 387 | 56501 | 246 | 56642 | 2.11484E-08 | 1.577096628 |
| CG_18 | 4543 | 52345 | 3522 | 53366 | 4.00753E-32 | 1.315051718 |
| CGG_13 | 444 | 56444 | 888 | 56000 | 6.64834E-35 | 0.496066898 |
| GGC_10 | 653 | 56235 | 938 | 55950 | 6.52738E-13 | 0.692633885 |

Contingency table used in Fisher’s exact test to assess the association between the presence or absence of a specific sequence motif (informative features) and the sgRNA. An odds ratio greater than 1 indicates that the feature is enriched in the sgRNA dataset, whereas an odds ratio less than 1 indicates it is depleted.

Odds Ratio = (a*d)/(c*b)

|  | a given sequence motif | other sequence motifs |
| --- | --- | --- |
| sgRNA dataset | a | b |
| Random DNA sequence of 20 bases dataset | c | d |

**Table S8.** Features identified by SVR*^WAA^-*based *DeepCC9* on the SpCas9-HF1 (SP) dataset. The numerical values following each feature indicate its presence or absence at a specific position on the sgRNA.

| Feature | Elements of contingency table | | | | P-value | Odds ratio |
| --- | --- | --- | --- | --- | --- | --- |
|  | a | b | c | d |  |  |
| AA | 72825 | 513348 | 69662 | 516508 | 3.97144E-19 | 1.051840121 |
| AA_2 | 4302 | 54315 | 3656 | 54961 | 6.74532E-14 | 1.190690973 |
| AA_3 | 4022 | 54595 | 3682 | 54935 | 6.43273E-05 | 1.099143866 |
| AA_10 | 3992 | 54625 | 3620 | 54997 | 1.09133E-05 | 1.110272319 |
| AA_11 | 4751 | 53866 | 3683 | 54934 | 1.42694E-33 | 1.315557419 |
| AA_12 | 4133 | 54484 | 3660 | 54957 | 3.10575E-08 | 1.139038367 |
| AA_14 | 3866 | 54751 | 3630 | 54987 | 0.005021829 | 1.069604435 |
| AA_16 | 4304 | 54313 | 3737 | 54880 | 6.04349E-11 | 1.163749415 |
| AAA_1 | 1885 | 56732 | 885 | 57732 | 4.98026E-84 | 2.167487455 |
| AAA_4 | 795 | 57822 | 929 | 57688 | 0.00124469 | 0.853775696 |
| AAA_12 | 1152 | 57465 | 858 | 57759 | 4.01798E-11 | 1.349526589 |
| AAAA | 4145 | 288945 | 3795 | 289293 | 8.01207E-05 | 1.093542071 |
| AACT_11 | 341 | 58276 | 244 | 58373 | 6.67852E-05 | 1.399867181 |
| AACG_12 | 122 | 58495 | 249 | 58368 | 3.69052E-11 | 0.488896075 |
| ATC_17 | 1081 | 57536 | 910 | 57707 | 0.000120645 | 1.191442625 |
| AC_2 | 2618 | 55999 | 3759 | 54858 | 4.95727E-49 | 0.682271162 |
| ACC_16 | 1466 | 57151 | 937 | 57680 | 8.61761E-28 | 1.579049692 |
| TAAC_13 | 165 | 58452 | 222 | 58395 | 0.004293782 | 0.742518463 |
| TTT_10 | 402 | 58215 | 911 | 57706 | 2.84325E-46 | 0.437415074 |
| TCTA_16 | 113 | 58504 | 214 | 58403 | 2.39343E-08 | 0.527125791 |
| TG_4 | 4112 | 54505 | 3651 | 54966 | 6.50818E-08 | 1.135792673 |
| TGTA_16 | 129 | 58488 | 235 | 58382 | 2.85563E-08 | 0.547941313 |
| CCAA_14 | 367 | 58250 | 268 | 58349 | 9.35106E-05 | 1.371730382 |
| CCAA_16 | 311 | 58306 | 246 | 58371 | 0.006512069 | 1.265637013 |
| CCAG_7 | 463 | 58154 | 226 | 58391 | 8.40061E-20 | 2.057021698 |
| GAGA_14 | 160 | 58457 | 220 | 58397 | 0.002389376 | 0.726526258 |
| GT_5 | 3443 | 55174 | 3676 | 54941 | 0.004548901 | 0.932660555 |
| GTAA_10 | 129 | 58488 | 241 | 58376 | 5.77697E-09 | 0.534244709 |
| GC | 73025 | 513145 | 69778 | 516392 | 4.8878E-20 | 1.053155384 |
| GCTG_2 | 431 | 58186 | 219 | 58398 | 5.5966E-17 | 1.975207047 |
| GCCC_13 | 299 | 58318 | 223 | 58394 | 0.000983707 | 1.342554514 |
| GGTG_4 | 354 | 58263 | 232 | 58385 | 4.94896E-07 | 1.529057153 |

Contingency table used in Fisher’s exact test to assess the association between the presence or absence of a specific sequence motif (informative features) and the sgRNA. An odds ratio greater than 1 indicates that the feature is enriched in the sgRNA dataset, whereas an odds ratio less than 1 indicates it is depleted.

Odds Ratio = (a*d)/(c*b)

|  | a given sequence motif | other sequence motifs |
| --- | --- | --- |
| sgRNA dataset | a | b |
| Random DNA sequence of 20 bases dataset | c | d |

**Table S9.** Features identified by SVR*^WAA^-*based *DeepCC9* on the WT-SpCas9 (WT) dataset. The numerical values following each feature indicate its presence or absence at a specific position on the sgRNA.

| Feature | Elements of contingency table | | | | P-value | Odds ratio |
| --- | --- | --- | --- | --- | --- | --- |
|  | a | b | c | d |  |  |
| AA | 68856 | 487187 | 66037 | 490003 | 2.71446E-16 | 1.048715054 |
| AA_1 | 5827 | 49777 | 3557 | 52047 | 1.62E-133 | 1.712884723 |
| AA_10 | 3780 | 51824 | 3442 | 52162 | 4.1034E-05 | 1.105361256 |
| AA_18 | 1760 | 53844 | 3523 | 52081 | 1.1303E-138 | 0.483216798 |
| AAA | 16785 | 372448 | 15514 | 373715 | 5.24581E-13 | 1.085606517 |
| AAA_3 | 1008 | 54596 | 881 | 54723 | 0.003445393 | 1.146815876 |
| AAA_15 | 949 | 54655 | 833 | 54771 | 0.006011774 | 1.141673663 |
| ATTG_7 | 157 | 55447 | 215 | 55389 | 0.003022657 | 0.729468703 |
| AGTG_3 | 343 | 55261 | 257 | 55347 | 0.000492398 | 1.33670737 |
| TAG_17 | 440 | 55164 | 914 | 54690 | 4.97886E-39 | 0.477263975 |
| TTAA_16 | 24 | 55580 | 242 | 55362 | 1.31595E-46 | 0.098784568 |
| TCTA_5 | 154 | 55450 | 216 | 55388 | 0.001457317 | 0.712165782 |
| CAAC_12 | 300 | 55304 | 225 | 55379 | 0.001186377 | 1.335141521 |
| CTC_17 | 1084 | 54520 | 841 | 54763 | 2.53183E-08 | 1.294686652 |
| CCCA | 5283 | 272737 | 3640 | 274380 | 3.96675E-69 | 1.460116873 |
| CGT_17 | 577 | 55027 | 879 | 54725 | 1.60748E-15 | 0.652825142 |
| CGG | 10320 | 378908 | 15476 | 373752 | 3.2448E-235 | 0.657764949 |
| CGG_11 | 574 | 55030 | 876 | 54728 | 1.40653E-15 | 0.651655179 |
| GA | 76725 | 479315 | 66122 | 489918 | 1.6445E-198 | 1.186023493 |
| GT_18 | 2485 | 53119 | 3456 | 52148 | 2.0034E-38 | 0.70589552 |
| GC_14 | 2426 | 53178 | 3450 | 52154 | 5.40568E-43 | 0.689647751 |
| GCCA_15 | 377 | 55227 | 217 | 55387 | 4.69494E-11 | 1.742360458 |
| GCGT_5 | 127 | 55477 | 225 | 55379 | 1.88166E-07 | 0.563447355 |

Contingency table used in Fisher’s exact test to assess the association between the presence or absence of a specific sequence motif (informative features) and the sgRNA. An odds ratio greater than 1 indicates that the feature is enriched in the sgRNA dataset, whereas an odds ratio less than 1 indicates it is depleted.

Odds Ratio = (a*d)/(c*b)

|  | a given sequence motif | other sequence motifs |
| --- | --- | --- |
| sgRNA dataset | a | b |
| Random DNA sequence of 20 bases dataset | c | d |

**Table S10.** Features identified by SVR*^GA^-*based *DeepCC9* on the eSpCas9 (1.1) (ES) dataset. The numerical values following each feature indicate its presence or absence at a specific position on the sgRNA.

| Feature | Elements of contingency table | | | | P-value | Odds ratio |
| --- | --- | --- | --- | --- | --- | --- |
|  | a | b | c | d |  |  |
| AA | 70793 | 498090 | 67504 | 501376 | 3.9564E-21 | 1.055641676 |
| AA_1 | 5944 | 50944 | 3643 | 53245 | 3.0829E-134 | 1.705318169 |
| AA_3 | 3917 | 52971 | 3581 | 53307 | 6.24141E-05 | 1.100766796 |
| AA_4 | 3185 | 53703 | 3511 | 53377 | 4.22711E-05 | 0.901642181 |
| AA_18 | 1796 | 55092 | 3596 | 53292 | 1.4052E-141 | 0.483125688 |
| AT_1 | 4989 | 51899 | 3609 | 53279 | 3.54402E-54 | 1.419134954 |
| ATAG_11 | 146 | 56742 | 213 | 56675 | 0.000468836 | 0.684636646 |
| ATCA_14 | 570 | 56318 | 230 | 56658 | 2.49305E-34 | 2.493222493 |
| ATCG_9 | 169 | 56719 | 225 | 56663 | 0.005442494 | 0.750369521 |
| ACAC_15 | 726 | 56162 | 236 | 56652 | 4.57698E-59 | 3.103110916 |
| ACC_17 | 2049 | 54839 | 927 | 55961 | 1.33567E-98 | 2.255579631 |
| AGAG_13 | 139 | 56749 | 214 | 56674 | 7.54405E-05 | 0.648674282 |
| TA_13 | 2871 | 54017 | 3510 | 53378 | 1.91787E-16 | 0.808272704 |
| TAGC_6 | 131 | 56757 | 190 | 56698 | 0.001154566 | 0.688756963 |
| TTT_9 | 384 | 56504 | 898 | 55990 | 2.42247E-48 | 0.423727023 |
| TGA_15 | 1123 | 55765 | 866 | 56022 | 6.68513E-09 | 1.302743056 |
| GAAC | 4805 | 279635 | 3689 | 280751 | 2.86194E-34 | 1.307719261 |
| GAGA_7 | 381 | 56507 | 232 | 56656 | 1.71642E-09 | 1.646571709 |
| GTAT_14 | 108 | 56780 | 216 | 56672 | 1.88358E-09 | 0.499048961 |
| GCAC_6 | 288 | 56600 | 210 | 56678 | 0.000531477 | 1.373318526 |
| GCCG_13 | 139 | 56749 | 225 | 56663 | 7.39671E-06 | 0.616841569 |
| GG_15 | 1920 | 54968 | 3578 | 53310 | 7.7449E-118 | 0.520426784 |
| GGA_13 | 661 | 56227 | 907 | 55981 | 4.30489E-10 | 0.7255877 |
| GGGC_15 | 88 | 56800 | 227 | 56661 | 2.41881E-15 | 0.386716511 |

Contingency table used in Fisher’s exact test to assess the association between the presence or absence of a specific sequence motif (informative features) and the sgRNA. An odds ratio greater than 1 indicates that the feature is enriched in the sgRNA dataset, whereas an odds ratio less than 1 indicates it is depleted.

Odds Ratio = (a*d)/(c*b)

|  | a given sequence motif | other sequence motifs |
| --- | --- | --- |
| sgRNA dataset | a | b |
| Random DNA sequence of 20 bases dataset | c | d |

**Table S11.** Features identified by SVR*^GA^-*based *DeepCC9* on the SpCas9-HF1 (SP) dataset. The numerical values following each feature indicate its presence or absence at a specific position on the sgRNA.

| Feature | Elements of contingency table | | | | P-value | Odds ratio |
| --- | --- | --- | --- | --- | --- | --- |
|  | a | b | c | d |  |  |
| AA | 72825 | 513348 | 69662 | 516508 | 3.97144E-19 | 1.051840121 |
| AA_1 | 6140 | 52477 | 3766 | 54851 | 2.9077E-138 | 1.704133468 |
| AA_10 | 3992 | 54625 | 3620 | 54997 | 1.09133E-05 | 1.110272319 |
| AA_11 | 4751 | 53866 | 3683 | 54934 | 1.42694E-33 | 1.315557419 |
| AA_13 | 3865 | 54752 | 3629 | 54988 | 0.005016395 | 1.069622343 |
| AA_14 | 3866 | 54751 | 3630 | 54987 | 0.005021829 | 1.069604435 |
| AA_16 | 4304 | 54313 | 3737 | 54880 | 6.04349E-11 | 1.163749415 |
| AAA_10 | 1228 | 57389 | 914 | 57703 | 8.06845E-12 | 1.350895972 |
| AAA_11 | 1176 | 57441 | 963 | 57654 | 3.66008E-06 | 1.225712137 |
| AAA_12 | 1152 | 57465 | 858 | 57759 | 4.01798E-11 | 1.349526589 |
| AAA_17 | 463 | 58154 | 910 | 57707 | 2.54443E-34 | 0.504880391 |
| AAAA_1 | 453 | 58164 | 228 | 58389 | 3.68856E-18 | 1.99452795 |
| ACCA_11 | 400 | 58217 | 250 | 58367 | 3.96156E-09 | 1.604122507 |
| TT_12 | 2185 | 56432 | 3641 | 54976 | 5.44193E-86 | 0.584626447 |
| TCTA_3 | 124 | 58493 | 233 | 58384 | 8.00507E-09 | 0.531197123 |
| CAAA_14 | 336 | 58281 | 229 | 58388 | 7.36718E-06 | 1.469942679 |
| CAGT_11 | 413 | 58204 | 233 | 58384 | 1.18207E-12 | 1.77801387 |
| CT_13 | 4679 | 53938 | 3737 | 54880 | 1.61885E-26 | 1.273940695 |
| CTA_4 | 471 | 58146 | 930 | 57687 | 1.98645E-35 | 0.502453723 |
| CTAA_6 | 134 | 58483 | 227 | 58390 | 1.08676E-06 | 0.589369658 |
| CGA_13 | 601 | 58016 | 943 | 57674 | 1.76858E-18 | 0.633570678 |
| GTG_14 | 591 | 58026 | 899 | 57718 | 9.57083E-16 | 0.653907667 |
| GCGT_3 | 136 | 58481 | 203 | 58414 | 0.000317733 | 0.669183196 |

Contingency table used in Fisher’s exact test to assess the association between the presence or absence of a specific sequence motif (informative features) and the sgRNA. An odds ratio greater than 1 indicates that the feature is enriched in the sgRNA dataset, whereas an odds ratio less than 1 indicates it is depleted.

Odds Ratio = (a*d)/(c*b)

|  | a given sequence motif | other sequence motifs |
| --- | --- | --- |
| sgRNA dataset | a | b |
| Random DNA sequence of 20 bases dataset | c | d |

**Table S12.** Features identified by SVR*^GA^-*based *DeepCC9* on the WT-SpCas9 (WT) dataset. The numerical values following each feature indicate its presence or absence at a specific position on the sgRNA.

| Feature | Elements of contingency table | | | | P-value | Odds ratio |
| --- | --- | --- | --- | --- | --- | --- |
|  | a | b | c | d |  |  |
| AA | 68856 | 487187 | 66037 | 490003 | 2.71446E-16 | 1.048715054 |
| AA_1 | 5827 | 49777 | 3557 | 52047 | 1.62E-133 | 1.712884723 |
| AA_10 | 3780 | 51824 | 3442 | 52162 | 4.1034E-05 | 1.105361256 |
| AA_11 | 4530 | 51074 | 3491 | 52113 | 1.99687E-33 | 1.324020033 |
| AA_18 | 1760 | 53844 | 3523 | 52081 | 1.1303E-138 | 0.483216798 |
| AAA_1 | 1799 | 53805 | 840 | 54764 | 3.30591E-81 | 2.179838925 |
| AAA_8 | 1062 | 54542 | 804 | 54800 | 1.86514E-09 | 1.327143754 |
| AG_18 | 3858 | 51746 | 3599 | 52005 | 0.001978314 | 1.07732985 |
| TATC | 2617 | 275403 | 3590 | 274430 | 1.75145E-35 | 0.726393907 |
| TAGC_12 | 121 | 55483 | 216 | 55388 | 2.45497E-07 | 0.559226016 |
| TCAG_6 | 348 | 55256 | 210 | 55394 | 5.12345E-09 | 1.661281516 |
| TCG_12 | 484 | 55120 | 933 | 54671 | 1.358E-33 | 0.514530978 |
| CACG_3 | 178 | 55426 | 232 | 55372 | 0.008653477 | 0.766493878 |
| CAG_15 | 1257 | 54347 | 873 | 54731 | 4.41183E-17 | 1.45003619 |
| CTT | 12968 | 376260 | 15424 | 373804 | 6.76404E-50 | 0.835279607 |
| CTT_16 | 267 | 55337 | 870 | 54734 | 1.13853E-75 | 0.30355234 |
| CCTA_11 | 165 | 55439 | 237 | 55367 | 0.000376357 | 0.695298356 |
| CGTT_16 | 97 | 55507 | 235 | 55369 | 2.142E-14 | 0.41173975 |
| GAGA_9 | 479 | 55125 | 249 | 55355 | 8.95823E-18 | 1.931721079 |
| GCAC_2 | 321 | 55283 | 253 | 55351 | 0.005007537 | 1.27033534 |
| GCTA_2 | 136 | 55468 | 234 | 55370 | 3.78197E-07 | 0.580169732 |
| GCG | 11422 | 377806 | 15556 | 373672 | 3.3314E-145 | 0.726216191 |
| GGG_14 | 288 | 55316 | 872 | 54732 | 2.37454E-69 | 0.326788341 |

Contingency table used in Fisher’s exact test to assess the association between the presence or absence of a specific sequence motif (informative features) and the sgRNA. An odds ratio greater than 1 indicates that the feature is enriched in the sgRNA dataset, whereas an odds ratio less than 1 indicates it is depleted.

Odds Ratio = (a*d)/(c*b)

|  | a given sequence motif | other sequence motifs |
| --- | --- | --- |
| sgRNA dataset | a | b |
| Random DNA sequence of 20 bases dataset | c | d |

**Table S13.** Sequence motifs associated with informative features that are enriched or depleted in footprint DNA

| Sequence motif | Elements of contingency table | | | | P-value | Odds ratio | 95% Confidence Interval (lower–upper) |
| --- | --- | --- | --- | --- | --- | --- | --- |
|  | a | b | c | d |  |  |  |
| AA/TT | 1680730 | 2712890 | 1335673 | 1781497 | 0 | 0.826324664 | 0.8239–0.8288 |
| AAA/TTT | 635760 | 2293320 | 547467 | 1530646 | 0 | 0.775077904 | 0.7719–0.7783 |
| AAAC/GTTT | 96142 | 2100668 | 72414 | 1486171 | 2.06977E-35 | 0.939294928 | 0.9301–0.9486 |
| AAC/GTT | 252332 | 2676748 | 176510 | 1901603 | 1.87913E-06 | 1.015582905 | 1.0091–1.0221 |
| ACAT/ATGT | 91548 | 2105262 | 63711 | 1494874 | 0.000135315 | 1.020311722 | 1.0098–1.0309 |
| ACCT/AGGT | 67533 | 2129277 | 42654 | 1515931 | 8.61421E-82 | 1.127206668 | 1.1133–1.1415 |
| ACGG/CCGT | 16719 | 2180091 | 10628 | 1547957 | 4.92597E-19 | 1.116973893 | 1.0888–1.1483 |
| TA | 546482 | 3847138 | 443170 | 2674000 | 0 | 0.857095364 | 0.8533–0.8612 |
| TAAT/ATTA | 100165 | 2096645 | 89165 | 1469420 | 0 | 0.787304303 | 0.7811–0.7923 |
| TACA/TGTA | 73138 | 2123672 | 53572 | 1505013 | 1.18624E-08 | 0.96751572 | 0.9592–0.9769 |
| TTAT/ATAA | 112938 | 2083872 | 102243 | 1456342 | 0 | 0.771967192 | 0.7666–0.7766 |
| TTCC/GGAA | 95769 | 2101041 | 65258 | 1493327 | 4.12941E-16 | 1.043065505 | 1.0345–1.0512 |
| TGAC/GTCA | 59493 | 2137317 | 37435 | 1521150 | 1.77701E-76 | 1.131074297 | 1.1170–1.1419 |
| TGGA/TCCA | 88730 | 2108080 | 57594 | 1500991 | 7.57242E-65 | 1.096943451 | 1.0856–1.1057 |
| CA/TG | 1256765 | 3136855 | 823161 | 2294009 | 0 | 1.11652889 | 1.1149–1.1225 |
| CAC/GTG | 260191 | 2668889 | 162419 | 1915694 | 0 | 1.149876148 | 1.1462–1.1592 |
| GT/AC | 875931 | 3517689 | 587320 | 2529850 | 8.3568E-306 | 1.072586774 | 1.0711–1.0789 |
| GTA/TAC | 186155 | 2742925 | 133892 | 1944221 | 7.97226E-05 | 0.985488983 | 0.9782–0.9893 |
| GCAC/GTGC | 50210 | 2146600 | 29715 | 1528870 | 2.5488E-140 | 1.203466264 | 1.1816–1.2179 |
| GG/CC | 1008841 | 3384779 | 675989 | 2441181 | 0 | 1.076348124 | 1.0715–1.0794 |
| GGA/TCC | 287398 | 2641682 | 189816 | 1888297 | 2.035E-143 | 1.082282635 | 1.0766–1.0874 |
| GGG/CCC | 286394 | 2642686 | 199054 | 1879059 | 1.17559E-13 | 1.023028797 | 1.0171–1.0274 |
| AAGA/TCTT | 113914 | 2082896 | 85388 | 1473197 | 1.12585E-35 | 0.943568644 | 0.9357–0.9477 |
| AGT/ACT | 273059 | 2656021 | 189144 | 1888969 | 4.15571E-17 | 1.026732366 | 1.0209–1.0309 |
| AGTG/CACT | 74959 | 2121851 | 47169 | 1511416 | 1.91578E-96 | 1.131973465 | 1.1223–1.1436 |
| AGCA/TGCT | 91548 | 2105262 | 55580 | 1503005 | 3.4592E-194 | 1.175938454 | 1.1664–1.1883 |
| TAAC/GTTA | 53393 | 2143417 | 38836 | 1519749 | 0.000159552 | 0.974798891 | 0.9625–0.9840 |
| TAG/CTA | 208420 | 2720660 | 151018 | 1927095 | 9.88719E-11 | 0.977551238 | 0.9732–0.9845 |
| TAGC/GCTA | 40869 | 2155941 | 26312 | 1532273 | 1.69149E-35 | 1.103924643 | 1.0901–1.1214 |
| CT/AG | 1226935 | 3166685 | 824267 | 2292903 | 0 | 1.07779077 | 1.0720–1.0797 |
| CCCT/AGGG | 83580 | 2113230 | 54478 | 1504107 | 9.42136E-56 | 1.091976209 | 1.0817–1.1003 |
| CG | 138559 | 4255061 | 96256 | 3020914 | 3.39655E-07 | 1.021973187 | 1.0165–1.0275 |
| CGAA/TTCG | 12190 | 2184620 | 7610 | 1550975 | 1.17629E-18 | 1.13722904 | 1.1044–1.1693 |
| CGAT/ATCG | 9345 | 2187465 | 5946 | 1552639 | 4.21293E-11 | 1.115536484 | 1.0757–1.1529 |
| CGCG | 7914 | 2188896 | 6524 | 1552061 | 3.10951E-19 | 0.860133281 | 0.8346–0.8884 |
| GA/TC | 1049319 | 3344301 | 718168 | 2399002 | 4.1748E-159 | 1.048109695 | 1.0439–1.0515 |
| GAAG/CTTC | 89199 | 2107611 | 58903 | 1499682 | 1.94765E-43 | 1.077534771 | 1.0649–1.0846 |
| GACC/GGTC | 42664 | 2154146 | 27619 | 1530966 | 3.09623E-33 | 1.097852592 | 1.0828–1.1133 |
| GTAA/TTAC | 61676 | 2135134 | 46144 | 1512441 | 2.33519E-18 | 0.946791318 | 0.9408–0.9586 |
| GCC/GGC | 239072 | 2690008 | 154166 | 1923947 | 5.4352E-205 | 1.109122825 | 1.1015–1.1158 |
| GCGG/CCGC | 27999 | 2168811 | 21871 | 1536714 | 9.04296E-27 | 0.907079225 | 0.8898–0.9184 |
| AT | 650148 | 3743472 | 507758 | 2609412 | 0 | 0.892531438 | 0.8912–0.8975 |
| ATT/AAT | 432452 | 2496628 | 360677 | 1717436 | 0 | 0.824795317 | 0.8229–0.8309 |
| ACCA/TGGT | 68014 | 2128796 | 43581 | 1515004 | 3.8564E-64 | 1.110659306 | 1.0968–1.1203 |
| TAA/TTA | 366362 | 2562718 | 311869 | 1766244 | 0 | 0.809632799 | 0.8075–0.8139 |
| TGC/GCA | 262736 | 2666344 | 160529 | 1917584 | 0 | 1.177075449 | 1.1716–1.1869 |
| CAAA/TTTG | 120158 | 2076652 | 88723 | 1469862 | 1.79039E-20 | 0.95858209 | 0.9477–0.9652 |
| CCAA/TTGG | 74218 | 2122592 | 49760 | 1508825 | 2.65986E-23 | 1.06023277 | 1.0718–1.0909 |
| CCCA/TGGG | 91501 | 2105309 | 60234 | 1498351 | 2.74772E-48 | 1.081139789 | 1.0691–1.0897 |
| GACT/AGTC | 53901 | 2142909 | 34837 | 1523748 | 3.96426E-43 | 1.100184428 | 1.0866–1.1134 |
| GAGA/TCTC | 88517 | 2108293 | 58636 | 1499949 | 1.24339E-39 | 1.07400889 | 1.0688–1.0883 |
| GGCC | 33548 | 2163262 | 22766 | 1535819 | 1.76779E-07 | 1.046190609 | 1.0287–1.0597 |

Contingency table used in Fisher’s exact test to assess the association between the sequence motifs associated with informative features and the footprint DNA (Figure 6). An odds ratio (OR) greater than 1 indicates that the sequence motif is enriched in nucleosomes, whereas an odds ratio less than 1 indicates it is depleted.

Odds Ratio = (a*d)/(c*b)

|  | a given sequence motif | other sequence motifs |
| --- | --- | --- |
| Nucleosomal DNA | a | b |
| Inter-nucleosomal DNA | c | d |

The 95% confidence interval (CI) for the odds ratio is calculated as:

$${CI}_{lower}= e^{\log\left( OR \right)-1.96 \times SE}$$

$${CI}_{lupper}= e^{\log\left( OR \right)+1.96 \times SE}$$

where the standard error (SE) is given by:

$$SE= \sqrt{\frac{1}{a}+\frac{1}{b}+\frac{1}{c}+\frac{1}{d}}$$

**Table S14.** Sequence motifs associated with informative features that are enriched or depleted in flanking linker DNA

| Sequence motif | Elements of contingency table | | | | P-value | Odds ratio | 95% Confidence Interval (lower–upper) |
| --- | --- | --- | --- | --- | --- | --- | --- |
|  | a | b | c | d |  |  |  |
| AA/TT | 718330 | 976352 | 499048 | 703289 | 1.18867E-50 | 1.036833664 | 1.033–1.043 |
| AAA/TTT | 272148 | 857640 | 200228 | 601330 | 1.03314E-45 | 0.952989643 | 0.946–0.960 |
| AAAC/GTTT | 38660 | 808681 | 27608 | 573560 | 0.396761293 | 0.993181268 | 0.974–1.009 |
| AAC/GTT | 100959 | 1028829 | 69179 | 732379 | 1.50999E-13 | 1.03887536 | 1.030–1.050 |
| ACAT/ATGT | 39650 | 807691 | 24998 | 576170 | 5.95403E-51 | 1.131470736 | 1.118–1.149 |
| ACCT/AGGT | 24775 | 822566 | 17372 | 583796 | 0.228618141 | 1.01217173 | 0.992–1.030 |
| ACGG/CCGT | 4719 | 842622 | 4288 | 596880 | 9.26042E-32 | 0.779559797 | 0.745–0.814 |
| TA | 258340 | 1436342 | 164826 | 1037511 | 1.5052E-292 | 1.132141698 | 1.126–1.139 |
| TAAT/ATTA | 48634 | 798707 | 32022 | 569146 | 1.00684E-26 | 1.082250349 | 1.066–1.096 |
| TACA/TGTA | 33434 | 813907 | 20750 | 580418 | 3.50918E-54 | 1.149043118 | 1.127–1.164 |
| TTAT/ATAA | 55402 | 791939 | 36846 | 564322 | 2.39195E-23 | 1.071446143 | 1.057–1.083 |
| TTCC/GGAA | 36401 | 810940 | 25593 | 575575 | 0.257188327 | 1.009497662 | 0.997–1.024 |
| TGAC/GTCA | 22354 | 824987 | 15161 | 586007 | 1.40959E-05 | 1.047328924 | 1.028–1.065 |
| TGGA/TCCA | 31933 | 815408 | 22933 | 578235 | 0.152428012 | 0.98743441 | 0.973–1.001 |
| CA/TG | 461546 | 1233136 | 330449 | 871888 | 2.82434E-06 | 0.987552683 | 0.983–0.993 |
| CAC/GTG | 91685 | 1038103 | 67265 | 734293 | 5.80755E-12 | 0.964135524 | 0.954–0.971 |
| GT/AC | 339343 | 1355339 | 234836 | 967501 | 3.65681E-25 | 1.03152013 | 1.025–1.036 |
| GTA/TAC | 83112 | 1046676 | 52248 | 749310 | 1.1696E-112 | 1.138789161 | 1.129–1.149 |
| GCAC/GTGC | 16740 | 830601 | 12464 | 588704 | 3.8659E-05 | 0.9519246 | 0.933–0.972 |
| GG/CC | 330164 | 1364518 | 270536 | 931801 | 0 | 0.83339052 | 0.829–0.838 |
| GGA/TCC | 101841 | 1027947 | 75772 | 725786 | 2.80594E-25 | 0.948968437 | 0.943–0.960 |
| GGG/CCC | 88357 | 1041431 | 78709 | 722849 | 0 | 0.779172562 | 0.772–0.786 |
| AAGA/TCTT | 50335 | 797006 | 32746 | 568422 | 1.86649E-36 | 1.09627902 | 1.083–1.110 |
| AGT/ACT | 112884 | 1016904 | 75260 | 726298 | 4.08676E-44 | 1.071280177 | 1.062–1.080 |
| AGTG/CACT | 28531 | 818810 | 19597 | 581571 | 0.000382528 | 1.034063016 | 1.015–1.050 |
| AGCA/TGCT | 31770 | 815571 | 22913 | 578255 | 0.053759395 | 0.983089102 | 0.970–0.999 |
| TAAC/GTTA | 23215 | 824126 | 15152 | 586016 | 4.92273E-16 | 1.089468265 | 1.070–1.107 |
| TAG/CTA | 92702 | 1037086 | 58683 | 742875 | 4.832E-113 | 1.131560471 | 1.123–1.144 |
| TAGC/GCTA | 16268 | 831073 | 10381 | 590787 | 1.33031E-17 | 1.114003948 | 1.088–1.136 |
| CT/AG | 466721 | 1227961 | 328270 | 874067 | 7.94755E-06 | 1.01201348 | 1.006–1.016 |
| CCCT/AGGG | 27005 | 820336 | 21607 | 579561 | 9.62703E-41 | 0.882992657 | 0.867–0.899 |
| CG | 41753 | 1652929 | 38525 | 1163812 | 0 | 0.763086323 | 0.754–0.777 |
| CGAA/TTCG | 4181 | 843160 | 3154 | 598014 | 0.009281075 | 0.940199108 | 0.901–0.982 |
| CGAT/ATCG | 3211 | 844130 | 2470 | 598698 | 0.002512114 | 0.922023148 | 0.873–0.971 |
| CGCG | 2343 | 844998 | 2446 | 598722 | 1.19718E-40 | 0.678711756 | 0.642–0.715 |
| GA/TC | 407944 | 1286738 | 283624 | 918713 | 2.14949E-21 | 1.02694529 | 1.022–1.031 |
| GAAG/CTTC | 34684 | 812657 | 23459 | 577709 | 7.57559E-09 | 1.051045543 | 1.033–1.069 |
| GACC/GGTC | 14152 | 833189 | 10994 | 590174 | 6.98407E-13 | 0.911797994 | 0.889–0.935 |
| GTAA/TTAC | 27511 | 819830 | 17466 | 583702 | 1.16165E-31 | 1.121450983 | 1.103–1.138 |
| GCC/GGC | 70565 | 1059223 | 62590 | 738968 | 0 | 0.786543279 | 0.775–0.799 |
| GCGG/CCGC | 8434 | 838907 | 8593 | 592575 | 3.8031E-124 | 0.693295357 | 0.669–0.720 |
| AT | 293238 | 1401444 | 190386 | 1011951 | 3.0109E-240 | 1.112164362 | 1.108–1.121 |
| ATT/AAT | 194286 | 935502 | 132141 | 669417 | 1.13318E-38 | 1.05209737 | 0.983–0.997 |
| ACCA/TGGT | 23989 | 823352 | 17520 | 583648 | 0.003112425 | 0.97060718 | 1.052–1.080 |
| TAA/TTA | 171625 | 958163 | 113898 | 687660 | 3.58922E-80 | 1.08143101 | 1.071–1.091 |
| TGC/GCA | 87664 | 1042124 | 66055 | 735503 | 4.92138E-34 | 0.936657116 | 0.928–0.950 |
| CAAA/TTTG | 46216 | 801125 | 34131 | 567037 | 7.64834E-09 | 0.958416883 | 0.947–0.973 |
| CCAA/TTGG | 26719 | 820622 | 19654 | 581514 | 9.4778E-05 | 0.963354804 | 0.807–0.837 |
| CCCA/TGGG | 28893 | 818448 | 24300 | 576868 | 1.03799E-87 | 0.838053455 | 1.007–1.043 |
| GACT/AGTC | 20188 | 827153 | 13980 | 587188 | 0.025891511 | 1.025126468 | 1.083–1.112 |
| GAGA/TCTC | 35212 | 812129 | 23238 | 577930 | 1.97532E-18 | 1.078306358 | 0.749–0.784 |
| GGCC | 9945 | 837396 | 9181 | 591987 | 1.85741E-74 | 0.765766014 | 0.751–0.781 |

Contingency table used in Fisher’s exact test to assess the association between the sequence motifs associated with informative features and the flanking linker DNA (Figure S1). An odds ratio (OR) greater than 1 indicates that the sequence motif is enriched in flanking linker regions, whereas an odds ratio less than 1 indicates it is depleted.

Odds Ratio = (a*d)/(c*b)

|  | a given sequence motif | other sequence motifs |
| --- | --- | --- |
| Flanking DNA of nucleosomal DNA | a | b |
| Flanking DNA of inter-nucleosomal DNA | c | d |

The 95% confidence interval (CI) for the odds ratio is calculated as:

$${CI}_{lower}= e^{\log\left( OR \right)-1.96 \times SE}$$

$${CI}_{lupper}= e^{\log\left( OR \right)+1.96 \times SE}$$

where the standard error (SE) is given by:

$$SE= \sqrt{\frac{1}{a}+\frac{1}{b}+\frac{1}{c}+\frac{1}{d}}$$

**Table S15.** Association between methylation at sgRNA genomic binding sites and Cas9 genome-editing efficiency

| Cas9 variant | Elements of contingency table | | | | P-value | Odds ratio |
| --- | --- | --- | --- | --- | --- | --- |
|  | a | b | c | d |  |  |
| WT | 3924 | 8698 | 1273 | 3528 | 2.9927e-09 | 1.25 |
| eSp | 2961 | 6403 | 2505 | 6510 | 1.3299e-08 | 1.20 |
| Sp | 3433 | 7554 | 1891 | 4979 | 1.1786e-07 | 1.19 |

Contingency table used in Fisher’s exact test to assess the association between methylation at Cas9 genomic binding sites and Cas9 genome-editing efficiency. An odds ratio greater than 1 indicates that methylation at sgRNA binding sites increases genome-editing efficiency, whereas an odds ratio less than 1 indicates reduced genome-editing efficiency.

Odds Ratio = (a*d)/(c*b)

|  | Methylated CpG | Unmethylated CpG |
| --- | --- | --- |
| sgRNA binding sites with genome-editing scores higher than the average | a | b |
| sgRNA binding sites with genome-editing scores lower than the average | c | d |


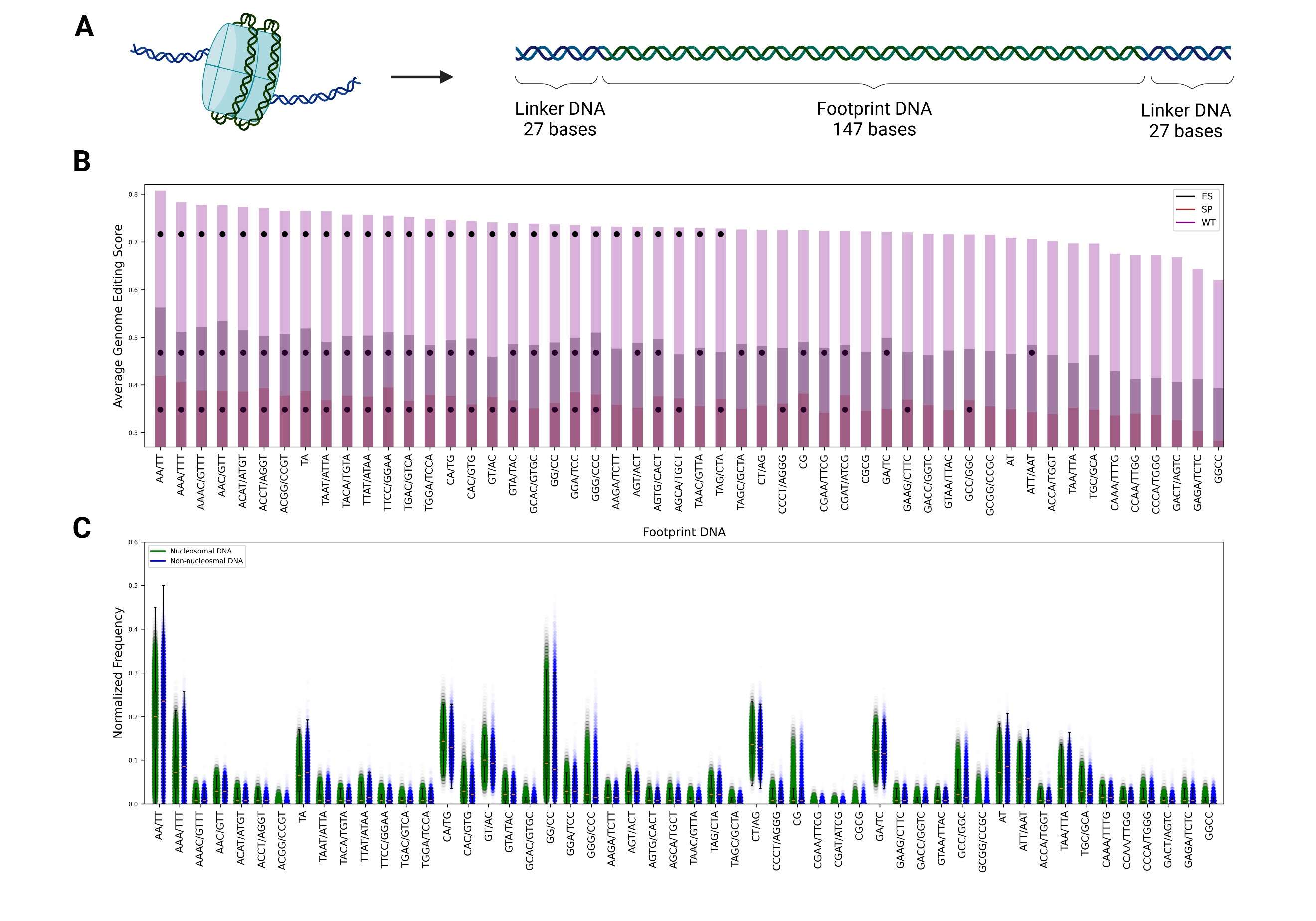


**Figure S1**. Enrichment or depletion of sequence motifs in nucleosomal DNA. (A) A schematic representation of a nucleosome, including histone proteins, the footprint DNA (147 bp), and the flanking linker DNA regions (27 bp). (B) Average Cas9 genome-editing scores of sgRNA sequences containing each sequence motif, illustrating how the motifs in part (C) were ranked. (C) Frequencies of specific sequence motifs in nucleosomal versus non-nucleosomal DNA. Green and blue indicate nucleosomal and non-nucleosomal DNA, respectively. Black, brown, and purple represent the ES, SP, and WT datasets, respectively. ES: eSCas9(1.1) dataset; SP: SpCas9-HF1 dataset; WT: WT-spCas9 dataset.


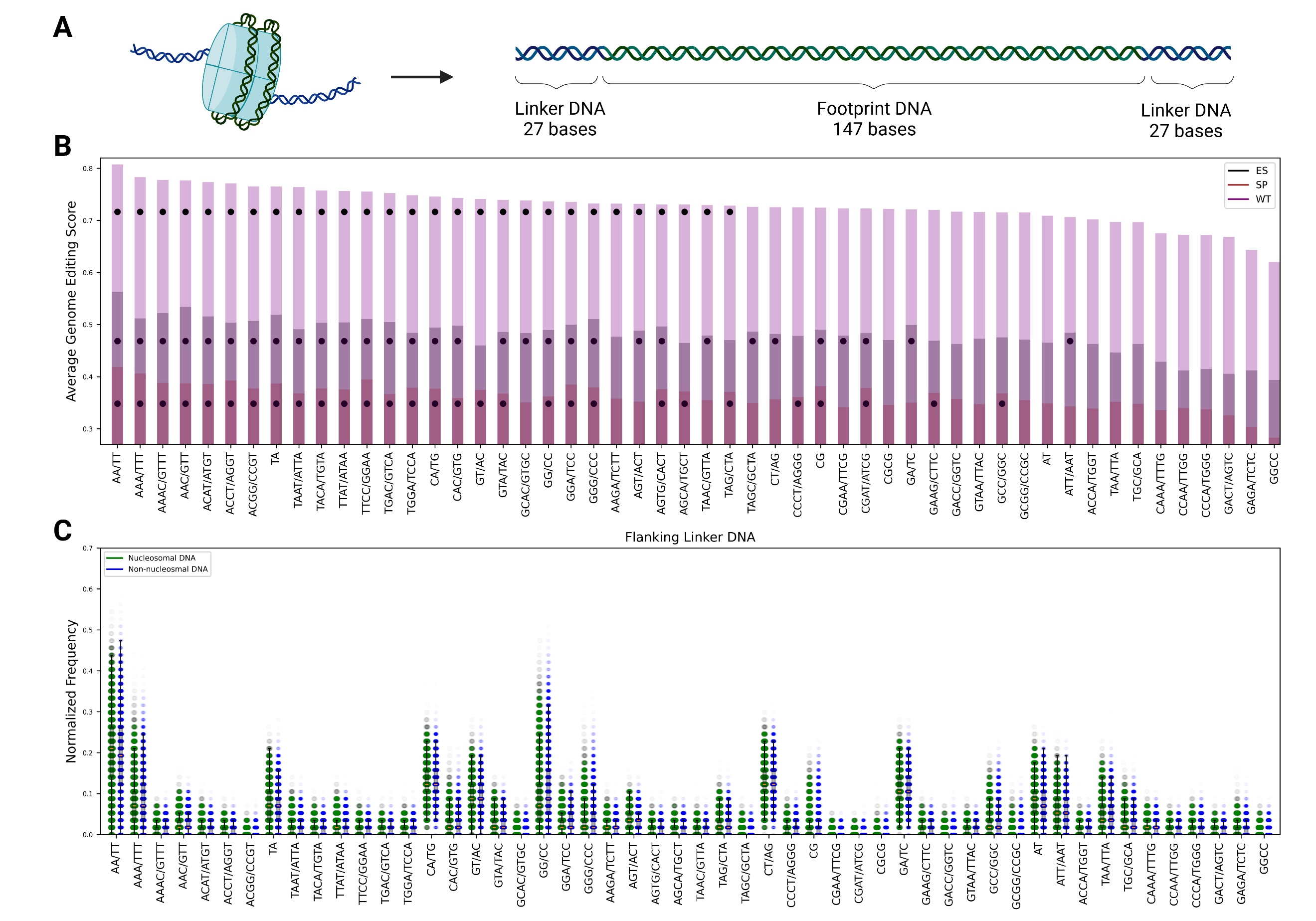


**Figure S2.** Enrichment or depletion of sequence motifs in flanking linker DNA regions. (A) A schematic representation of a nucleosome, including histone proteins, the footprint DNA (147 bp), and the flanking linker DNA regions (27 bp). (B) Average Cas9 genome editing scores of sgRNA sequences containing specific sequence motif, illustrating how the motifs in part (C) were ranked. (C) Frequencies of specific sequence motifs in the flanking DNA of nucleosomal versus non-nucleosomal DNA. Green and blue indicate flanking DNA from nucleosomal and non-nucloesomal DNA, respectively. Black, brown, and purple represent the ES, SP, and WT datasets, respectively. ES: eSpCas9(1.1) dataset; SP: SpCas9-HF1 dataset; WT: WT-SpCas9 dataset.
